# Child development, physiological stress and survival expectancy in prehistoric fisher-hunter-gatherers from the Jabuticabeira II shell mound, South Coast of Brazil

**DOI:** 10.1371/journal.pone.0229684

**Published:** 2020-03-11

**Authors:** Luis Pezo-Lanfranco, José Filippini, Marina Di Giusto, Cecília Petronilho, Veronica Wesolowski, Paulo DeBlasis, Sabine Eggers

**Affiliations:** 1 Laboratório de Antropologia Biológica, Departamento de Genética e Biologia Evolutiva, Instituto de Biociências da Universidade de São Paulo, Cidade Universitária USP, São Paulo, Brazil; 2 Museu de Arqueologia e Etnologia da Universidade de São Paulo, Cidade Universitária USP, São Paulo, Brazil; 3 Naturhistorisches Museum Wien, Anthropologische Abteilung, Vienna, Austria; Museo delle Civiltà, ITALY

## Abstract

In this study, we shed light on the interdependency of child growth, morbidity and life expectancy in the fisher-hunter-gatherers of the Jabuticabeira II shell mound (1214–830 cal B.C.E. - 118–413 cal C.E.) located at the South Coast of Brazil. We test the underlying causes of heterogeneity in frailty and selective mortality in a population that inhabits a plentiful environment in sedentary settlements. We reconstruct osteobiographies of 41 individuals (23 adults and 18 subadults) using 8 variables, including age-at-death, stature, non-specific stress markers (cribra orbitalia, porotic hyperostosis, periosteal reactions, periapical lesions and linear enamel hypoplasia), as well as weaning patterns based on stable isotope data to examine how stress factors module growth and survival. Our results show that shorter adult statures were linked to higher morbidity around weaning age and higher chances of dying earlier (before 35 years) than taller adult statures. In addition, short juvenile stature was related to physiological stressors and mortality. The adult “survivors” experienced recurrent periods of morbidity during childhood and adulthood, possibly associated with the high parasite load of the ecosystem and dense settlement rather than to malnourishment. An association between early-stress exposure and premature death was not demonstrated in our sample. To explain our data, we propose a new model called “intermittent stress of low lethality”. According to this model, individuals are exposed to recurrent stress during the juvenile and adult stages of life, and, nevertheless survive until reproductive age or later with relative success.

## Introduction

The examination of child growth, health, nutritional status, and life expectancy of ancient populations is among the main concerns of bioarchaeology. The study of juveniles in different contexts of population growth and subsistence is of first importance to assess the balance between nutrition, disease, and death, because population growth theoretically depends, among other factors, on low infant-mortality and long enough survival until reproductive age [1,2]. Therefore, studying juveniles is also useful to test how morbidity in early life may impact frailty and survival in later life [3,4]. This applies especially to nutritional and demographic transitions. Sedentism and the adoption of agriculture, for example, lead to a decrease in life expectancy and poor health conditions through population growth and malnutrition [5,6].

Bioarchaeological inferences about health in the past are modulated by the concepts of “heterogeneity in frailty” and “selective mortality”, under the interpretative limits imposed by the “Osteological Paradox” [7]. A key question to understand these concepts is: does a skeleton without detectable bone lesions represent a healthy person or a fragile individual who perished at a first exposure to disease? “Heterogeneity in frailty” suggests that variable susceptibility to disease from individuals do not allow reliable inferences about the populations’ health, since the factors involved (i.e. genetic, socioeconomic, environmental, chronologic, etc.) are not easily identifiable. “Selective mortality” means that skeletal lesions may not accurately reflect a living population at risk of death at any age. This is because deceased individuals integrate the age structure of the sample according to different morbidity experiences, frailty and death reasons [7–9].

Documenting the heterogeneity in frailty and selective mortality is critical to discussions about health in the past. Recent reviews have highlighted the importance of analyzing data within its historical/archeological context in order to examine causes, effects and correlations between heterogeneity in frailty and selective mortality [9]. In recent years, bioarcheological researches introduced new approaches to assess the relation between early-stress, later morbidity and mortality [10–14]. Focusing on the study of health conditions in past foraging societies, two hypotheses were tested: the “plasticity/constrain” model and the “predictive adaptive response” model. The first predicts that people trade-off in future growth and maintenance when early investment in growth and survival is required, thus, individuals with more stress episodes during childhood are more prone to higher morbidity and early mortality in adulthood [4,15,16]. The second suggests that processes by which organisms adapt to their environment can operate during early development and induce adjustments, by epigenetic mechanisms, in the mature phenotype. Individuals who experienced early-life stressors can improve their physiological competence to deal with future stress events [4,17].

The prehistoric shell mound builders from the Brazilian South Coast provide an unparalleled opportunity to test these models and discuss differences in the underlying causes of heterogeneity in frailty and selective mortality between fisher-hunter-gatherers around the world. The current archaeological evidence suggests that the groups who built the Brazilian shell mounds (*sambaqui*) lived for thousands of years in sedentary or semi-sedentary settlements located in plentiful environments (lagoons, mangroves or estuaries), with an abundant and stable marine-based subsistence, and experienced high population growth [18]. However, despite this “idyllic” context of affluence, *sambaqui* populations have shown high frequencies of non-specific stress markers [19,20] that suggest other biocultural factors involved.

### Jabuticabeira II: Lifestyle, diet and health

**Fig 1:**
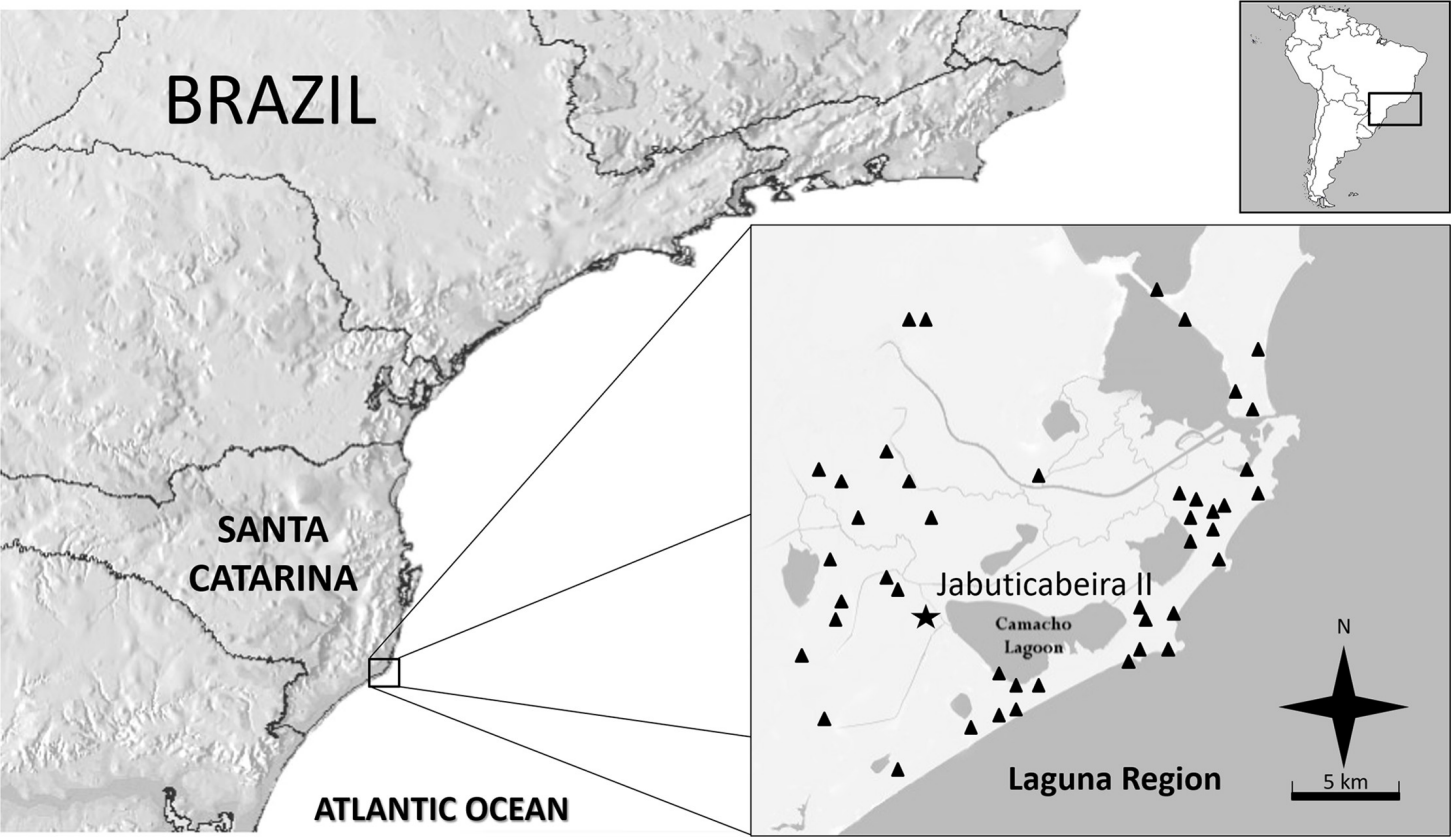
Setting of Jabuticabeira II. The star represents Jabuticabeira II, the triangles represent other *sambaquis* mapped for the Laguna Region (modified from Boyadjian et al. [31:137]).

Jabuticabeira II (UTM 22J - 0699479E; 6835488S) is located in the Laguna region, the highest density area of *sambaqui*s from the Brazilian Southern coast (Fig 1; [21]). Jabuticabeira II is a medium size shell mound (400 x 250 x 10 m in height), settled on a paleodune, 3 km from *Laguna do Camacho*, one of several water sources associated with a barrier-lagoon geological system formed during the Holocene [22,23]. The predominant ecosystems in the region are *restingas* and dense ombrophilous forests rich in fauna, and subtropical weather [24]. Radiocarbon dating indicates a long occupation period between 1214–830 cal B.C.E. and 118–413 cal C.E. [21:35; 25:260, 296].

Jabuticabeira II is one of 65 *sambaquis* mapped (located at equidistance of about 5 km, some of them higher than Jabuticabeira II) around the lagoon system. This large number of settlements and their overlapping occupation history attests to a fairly dense occupation between 7500 and 900 cal BP, with groups of *sambaquis* interacting intensely in this stable and plentiful area [26].

The analysis of faunal macroremains and stable isotopes in the site demonstrates higher reliance on fishing than mollusks gathering, while some females exhibit diets of lower trophic level than most adults [25,27]. Osteological activity markers also point to fishing as the most remarkable feature of the Jabuticabeira lifestyle with some sex-related differences [28,29]. Anthracological analyses, microremains in dental calculus and oral pathology studies support the idea of regular plant consumption at the site [30–32]. Nevertheless, its impact on nutrition and health is still to be unraveled.

Stratigraphic studies suggest that Jabuticabeira II was formed by the incremental accumulation of funerary areas [21]. A total of 204 burials and 282 individuals were excavated from different areas at the site. Between 2000- and 1500-years BP, several *sambaquis* of the Laguna region, including Jabuticabeira II, experienced a change in their depositional pattern. The predominant deposition of shells was replaced by the accumulation of a black superficial layer (0.50–1.50 m of thickness), composed mainly of intentionally burned fish bones and charcoal [33]. This depositional change (from shell-rich layers to fish-rich-layers) was not related to variations in diet, technology or constructive patterns [21,26,33]. Biodistance studies also did not detect biological differences among groups [34].

Our former bioarchaeological study on *sambaqui* dwellers at the Brazilian coast show that Jabuticabeira II people had shorter stature than several Amerindian groups [35], as well as high prevalence of bone lesions associated with non-specific physiological stress and infectious processes. These conditions were attributed to sedentary-dense settlements, as well as ecological factors [19–36].

### Osteobiographic approach and life-history

In theory, an osteobiographic approach, a context-driven analysis of human remains of given individuals [37–39], can reveal life course processes of exposure to situations of homeostatic imbalance or stress and their impact. Integrated analyses of non-specific physiological stress markers, stature and weaning behaviors, allow the assessment of past health conditions to test hypotheses about morbidity, frailty and increased risk of mortality in ancient populations [4,13,40].

The study of growth can be particularly informative about adaptive responses of the body to the environment. The expression of stature is modulated by a complex interaction of genetic, socioeconomic and cultural factors [6,41,42]. According to available genomic data and several studies in twins from affluent societies, the expression of stature is highly controlled by genes. The estimated heritability ranges between 0.68 and 0.93 (with higher values in men, and lower values in poor populations: [43–45]). Apart from genetic factors, several environmental conditions can modulate height expression. The influence of those conditions on heritability rates is highest in early childhood, decreasing during adolescence and early adulthood [46].

The relevance of nutrition for linear growth has been widely recognized, and the mean height of a population can be considered a proxy for its nutritional condition [47–49]. Cumulative evidence suggests that patterns of development (in height and weight) of well-nourished and healthy children are similar during the first five years of life regardless of ethnic origin [50]. Irreversible retardation of child growth is associated with chronic malnutrition and recurrent infectious diseases, such as diarrhea, through negative effects on the metabolism, especially in contexts of population agglomeration, lack of resources and social stratification [40,51–54].

In contemporary populations of developing countries, subject to nutritional (mostly protein) deficiencies, mean height in children and adults is substantially lower than in developed countries [49,55,56]. Additionally, it is widely believed that the duration and intensity of factors that produce physiological stress may also have direct and indirect consequences on individual development and premature death [49,57,58]. In bioarchaeology, numerous authors have shown that short height-for-age (*stunting*) is a signal of poor health, being commonly associated with increased risk of morbidity and early mortality [4,13,40,42]. Therefore, a relative short adult stature is almost always associated with stress factors during the growth period (including the intrauterine period) and should undergo a detailed contextual analysis [10,12,59].

In this sense, weaning habits and juvenile diets influence morbidity and mortality, especially at the more vulnerable ages during childhood. Adequate breastfeeding and weaning patterns are positively related with child health, growth and increasing chances of survival [60], whereas poor breastfeeding habits result in arrested growth, fragile health and increased infant mortality [61,62]. The early cessation of breastfeeding and the introduction of supplementary diet or adult diet, eventually in association with pathogens, makes weaning age an especially vulnerable period [53,63].

Stature is an informative marker of environmental insults experienced during the earlier phases of growth (in terms of occurrence, duration and timing) and can be contrasted to other markers to uncover whether individual growth (normal or delayed) is related to an individual's exposure to situations of non-specific physiological stress. Physiological stress markers currently used in bioarchaeology such as enamel hypoplasia, cribra orbitalia, porotic hyperostosis, and periosteal reactions provide an overview of health disorders at certain ages [14,64]. Below we shortly describe these osteological markers.

Linear enamel hypoplasias (LEH) are horizontal defects located on the labial and buccal surface of the dental crown. Although other kinds of hypoplastic defects can occur (pits, vertical grooves, areas of missing enamel), LEH are the most common [54,65]. LEH is associated with disturbances in the perikymata. Perikymata are incremental layers of enamel (visible as “concentric ripples” on the tooth crown surface and extend horizontally around the circumference of the crown), the outer expression of striae of Retzius, underlying incremental growth layers. LEH result from accentuated perikymata during the enamel formation process followed by recovery periods [65]. The average secretion rate or periodicity of perikymata and striae of Retzius is 8–9 days. Therefore, the analysis of LEH allows evaluating the timing, duration and severity of short-term non-fatal stress events [66–69].

LEH have been used to infer metabolic insults related to multiple non-specific factors, including systemic infectious diseases and nutritional stress during childhood [10,13,70–72]. Although caries and dental attrition can destroy crowns affected by LEH, the enamel defects acquired during development periods do not remodel. Thus, LEH recorded in adults provide a record of metabolic stress during juvenile periods [65,69,70]. Such information can be contrasted to mortality profiles to test if exposure to child stress has a negative impact on survival rates, as well as other issues concerning heterogeneity in frailty and selective mortality [10].

Cribra orbitalia (CO) and porotic hyperostosis (PH) appear as increasing porosity in the orbital roof and the cranial vault, respectively. They are thought to be the effect of bone marrow expansions linked to the compensatory production of blood cells in response to increased physiological demand and have been mostly associated with non-specific anemia [64,73,74]. These two entities are not necessarily related, and a variety of newer studies indicate that they may have different etiologies such as local inflammation, scurvy, rickets [74] or different types of anemia [75]. These porosities are more common in coastal settings, possibly due to limited access to parasite–free water and frequent contact with chronic parasitic diseases associated with population growth, dense settlements and poor sanitation [54].

Periosteal Reactions (PR) are new bone formations associated with augmented activity of osteoblasts and osteoclasts in response to several stimuli over the periosteum. Their etiologies include trauma, local or systemic inflammation or infection, nutritional deficiencies [64,76–79], neoplasia and metabolic diseases [80]. Bone markers of specific infection include osteomyelitis, signs of bone remodeling and diagnostic patterns of localized and diffuse periosteal reactions typical of systemic infectious processes [14,64]. Several studies support that sedentism, population growth and aggregation are strongly related to increased prevalence of infections and periosteal reactions [54].

Finally, many clinical and epidemiological studies showed bi-directional relationships between poor oral health (mainly periodontal disease and oral infections) and chronic/systemic diseases with potential effects on mortality [81,82]. Periapical lesions (PL) located in mandible and maxilla are potential sources of infection in joints, heart and kidneys (and vice versa) and are indirectly related to immunological depletion and other shared risk factors [83–85]. Periapical lesions result from acute or chronic infections (abscesses and granulomas, respectively) in alveolar bone, associated with caries, periodontal disease, pulp exposures due to severe occlusal wear, and also from sinus infections [86].

### Aims and expectations

In this study we evaluate child stunting, malnutrition and morbidity as modulating factors for development and survival in the Jabuticabeira II *sambaqui* using several osteobiographic markers. These markers include adult and juvenile height, linear enamel hypoplasias, cribra orbitalia, porotic hyperostosis, periosteal reactions, and age-at-death. Finally, data from our earlier isotopic weaning study are used herein to evaluate the implications of nutrition during juvenile ages on morbidity [87].

Our aims are to identify periods of higher vulnerability and causes of frailty among Jabuticabeira II individuals and to examine how stress factors influence them, to answer the following questions: Is there evidence of early stress periods in Jabuticabeira II individuals? How does early-life exposure to physiological stress influence individual health and mortality risk in adulthood?

More specifically, six hypotheses about child development, health and survival expectations in Jabuticabeira II are tested: 1) higher early-stress factors during juvenile periods (presence, duration, and timing of LEH in relation to weaning) are associated with shorter adult stature; 2) higher early-stress factors during juvenile periods (presence, duration, and timing of LEH in relation to weaning) are associated with earlier adult age at death (<35 years); 3) higher juvenile morbidity (CO, PH, PR and LEH) is associated with higher juvenile mortality (>1 year and <1 year); 4) higher adult morbidity (CO, PH, PR, PL) is associated with earlier adult age at death (<35 years); 5) infant malnutrition (no breastfeeding or early weaning age) is associated with higher morbidity (CO, PH, PR and LEH) in juveniles >1 year; 6) infant malnutrition is associated with higher morbidity (CO, PH, PR and PL) in adults.

## Materials and methods

The Jabuticabeira II collection is housed in the *Museu de Arqueologia e Etnologia da Universidade de São Paulo*, Brazil. No permits were required for the described study, which complied with all relevant regulations. As in the case of other archaeological contexts, this collection is seriously compromised by taphonomic damage. Due to the high degree of bone fragmentation, only 41 individuals (adults and juveniles), from a total of 282 evaluated records, met the following inclusion criteria: a) one or more long bones in good condition for measurements; b) availability of alveolar bone and teeth in order to assess dental age, enamel hypoplasia, and maxillary infections; c) previously studied isotopic values that allow for the evaluation of the weaning age and/or infant/adult diet estimations [87]. Because the focus of this work is child development, the first criterion was imperative.

### Estimation of sex and age at death

Adult sex was estimated according to morphological characteristics of the skull and pelvis [88]. Due to the difficulties of sex determination in juveniles, all of them were classified as “undetermined” [89]. The age at death in juveniles was estimated based on dental formation-eruption sequences published by Ubelaker [90], based on Arikara Indians of North America, and by Gaither [91], based on indigenous populations from Central Andes. The degree of epiphyseal union and vertebral development [89] were also observed to confirm our estimations. Age at death in adults was taken from our previous research 19,32] also based on standardized morphological markers [88]. Adults were classified into broad categories: Young Adult (YA) = 20–35 years; Middle Adult (MA) = 35–50 years; Old Adult (OA) > 50 years; Adult = Age not determined). Because a detailed demographic study in Jabuticabeira II is missing and the mean age-at-death of Jabuticabeira II individuals is unknown, the age of 35 years, was arbitrarily chosen as the cut-off to differentiate “early” from “late death”, assuming that Young Adults (<35 years) died at an unexpected early age.

### Osteometric recording

The osteometric recording was based on Buikstra and Ubelaker ([88]: 46, 79–84) standards. All complete bones from either side were measured with a sliding digital caliper (Mitutoyo 150 mm) and a standard osteometric board by one of us (LPL). In cases of well-preserved bones on both sides, only left bones were considered for analysis [92]. Pathological long bones were excluded from this study.

### Stature estimation in adults

Stature estimation in adults was based on regression equations from the measurement of long bones. Ideally, formulae should be developed on a subset of complete individuals from the same population and then applied for stature estimation in the rest of the sample. However, because our sample is inappropriate for that, other formulae that respect the allometric variation in the population were selected. A rather straightforward approach to evaluate similarities in body proportions between the population used for developing the method and the subject population is to test the consistency of results obtained with formulae based on different skeletal elements using the Delta of Gini method ([6,93], S1 File). This approach was used herein to decide which of two available methods should be used for Jabuticabeira II.

Between Pomeroy and Stock ([94], conceived from Andean archaeological populations), and Genovés ([95], corrected by Del Angel and Cisneros [96], based on Mesoamerican individuals), the first method provided more consistent Delta of Gini estimates (DG = 1.65 vs. DG = 2.37) in 4 individuals with complete set of long bones. Thus, we used the formulae by Pomeroy and Stock ([94]: Table 6), the most appropriate for our sample (S1 File).

When it was not possible to use the tibia and/or the femur measurements, height was estimated using any available long bone; we also calculated Z-scores (using the median group values of males and females from Jabuticabeira II) to analyze correlations between stature and other markers.

Adult height is the outcome of a complex process. Considering the high heritability of adult eight expression, classifying any adult as stunted or non-stunted based on bone measures is virtually impossible. We can only know if they were relatively smaller or taller than others. Conventionally, “short stature” is defined as a height ≤ -2 SD (standard deviations) of a specific population [45]. However, due to the small sample size, we were compelled to classify adult individuals by relative stature using the median of each group as classification parameter, arbitrarily. Thus, relative short stature in adults was classified as “below the median”.

### Stature estimation in juveniles

**Fig 2:**
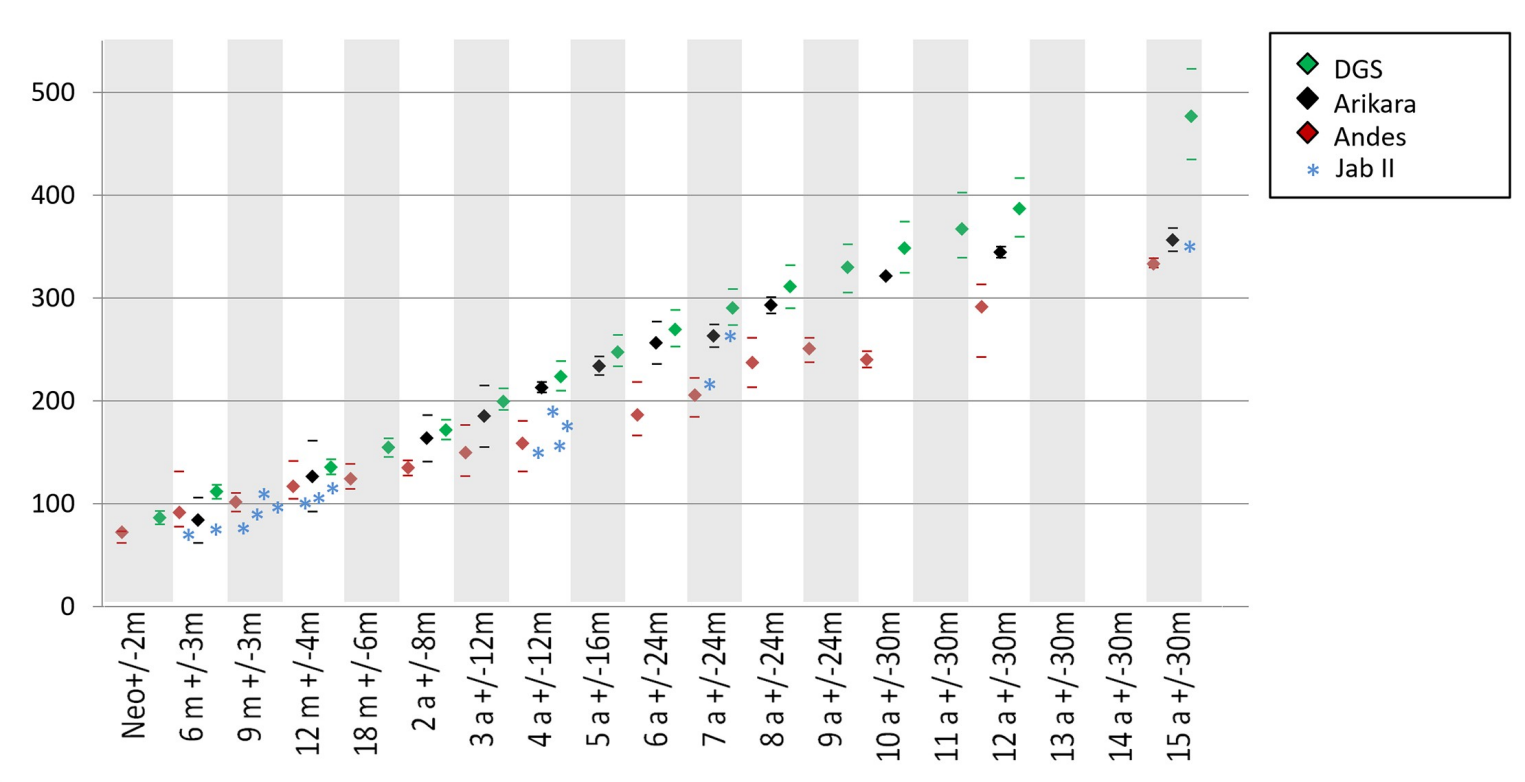
Femoral lengths by age in juveniles from Jabuticabeira II and reference populations. Asterisks represent the individual values of Jabuticabeira II juveniles. For the compared populations marks represent mean values and the lines represent the range of variability. DGS: Denver Growth Study.

As our goal was assessing if juvenile growth is compatible with stunting three strategies were applied:

1. Height estimation in juveniles between 1–17 years old was based on the long bone’s regression formulae postulated by Ruff ([92]: Tables 4 and 5) based on the maximum diaphyseal length (without epiphysis) that is applicable to undetermined-sex juveniles. Stunting was diagnosed as height by age Z-scores (HAZ) less or equal to two (≤ -2 SD, moderate) or three (≤ -3 SD, severe) standard deviations. The Z-scores were calculated using the simplified formula proposed by the WHO [50]: *Z = [(X—M)/SD]*, where *X* is the measured or estimated stature, *M* is the median and *SD* the standard deviation for each age-range (values provided in the *Height-for-age* tables of Child Growth Standards, [50]). For individuals <5 years old the calculation was performed by applying the package Anthro v.3.2.2 [97]. Considering that juvenile sex is unknown, and the WHO charts are divided by sex, we calculated HAZ for males and females.
2. The WHO growth charts [50], however, do not include long bone measurements (and therefore are not directly applicable to archaeological material). Thus, a second strategy to evaluate stunting consisted of comparing long bone lengths of each dental-age cohort to those of contemporary children from the *Denver Growth Study* [98]. This is considered a good parameter of normal growth in extant [99], as well as, archaeological juveniles from North America [90] and the Central Andes [100]. However, this method only aims to detect differences by age in the length of bones between populations. Because our juvenile individuals were not sexed and the *Denver Growth Study* tables are categorized by sex, we calculated the arithmetic mean and a range of variation of the entire sample (merging girls and boys) for each age-cohort, in order to create a comparative parameter for each bone. As the tables by Maresh [98] only show average and values between 90th and 10th percentiles, “probable stunting” was arbitrarily attributed to diaphyseal lengths below the 10th percentile [99]. The diaphyseal measures of Andean individuals [100:37,74–79] are not divided by sex because they correspond to non-sexed juveniles of archaeological origin.
3. Another strategy to detect stunting was to calculate height from the length of upper and lower limbs. We expect upper limbs to provide more reliable height reconstruction according to dental age, because there are less sensitive to environmental conditions due to their slower growth [101,102]. In individuals with probable stunting, the estimated height based on lower limbs should be shorter than the estimated height based on upper limbs.
4. Finally, as our goal is to assess if Jabuticabeira II growth patterns are compatible with stunting, the percentage of adult femur length attained at different ages was calculated following the method suggested by Humphrey [103:Fig 2B] to avoid potential source of error using regression formulae and standards of other populations. The percentage was calculated using the children’s femur lengths divided by the average adult femur length (including epiphyses) of the pooled sample.

### Weaning and infant malnutrition

The dietary data from Jabuticabeira II (approximate ages at the beginning and the end of weaning, supplementary and post-weaning diets) originates from nitrogen and carbon isotope values of dentin sections from adults, and bones from juveniles previously published [27,87]. These studies revealed an age range of weaning between 2 and 3 years (most likely 2.3 years old). According to those studies child diet was similar to adult diet, both concerning broad items as well as proportions, from around the age of 1.5 years on [87]. Breastfeeding and the approximate ages at the start and end of weaning were inferred from δ^15^N and δ^13^C values for each individual (S1 File). Malnourished individuals were diagnosed according to the following criteria: absence of signs of breastfeeding (δ^15^N and δ^13^C below the expected value for breastfeeding, around 2–3‰ above the mean value of women) and early weaning age (below 2 years).

#### Morbidity

The evaluation of morbidity included the recording of non-specific physiological stress markers:

Linear enamel hypoplasia (LEH) was observed directly under natural and artificial light with the help of an angled dental explorer and 10x magnifying glass [70]. In individuals with complete dentition every tooth was observed in order to record the LEH presence. In individuals with incomplete dentition, only incisors and canines were analyzed. Only cases with both antimeres affected by LEH were considered positives [88]. LEH was recorded in terms of presence/absence and quantity of affected teeth per individual. Teeth with carious lesions were not considered for analysis.

The approximate age of occurrence of LEH (in years and months) was estimated according to Reid and Dean [67], taking into account the loss of coronal height due to dental wear (only teeth with more than 70% of coronal height were considered). A “LEH incidence period”, that means the period of recurrent LEH suffered by one individual, was calculated as the time between the first and the last hypoplasia of a given individual.

Cribra orbitalia (CO) and porotic hyperostosis (PH), active or healed, were recorded in terms of presence/absence [88]. Localized and diffuse periosteal reactions and signs of acute or chronic bone infection, such as osteomyelitis, bone remodeling and fistula (all considered herein as PR) were recorded in terms of presence/absence in all available bones [64]. Periapical lesions (PL) of mandible and maxilla were recorded as presence/absence and number of lesions per individual [32].

All conditions were recorded by macroscopic inspection, aided by the use of 10x magnification lens, in each individual. Statistical analyses to test differences in the prevalence of markers between groups were considered unnecessary since this subject is a subsample of an entire population analyzed in other bioarchaeological studies earlier [19,32,35,104]. Furthermore, since the choice of the skeletons is based on their preservation, they may represent an age and sex-biased sample. To examine bivariate correlations, the Spearman's coefficient (ρ) at p<0.05 was used. For some frequency comparisons, we used Student’s t-test, Chi-square, and Mann Whitney U. To detect significant interactions between more than two variables or predictor variables we used the binary logistic regression analysis [40]. The operationalization of markers in dichotomous variables appears in Table 1. All statistical analyses were performed using SPSS 18.0 (IBM^®^).

**Table 1:** Operationalization of stress markers in dichotomous variables for logistic regression analysis.

Dichotomous variable	Infant malnutrition^1^		Infant morbidity		Juvenile short height-by-age (stunting)	Adult relative short height	Adult morbidity	Early mortality	
	Juveniles^2^	Adults^3^	Juveniles	Adults	Juveniles	Adults	Adults	Juveniles	Adults
No (0)	Breastfed: δ^15^N > 2‰ above the female mean value and/or Mean weaning-age: 2–3 years old		No stress markers	No LEH	Long bone length into/above the range of variability and/or HAZ > -2 SD	Stature above the group median (in M, F) or Z-score > 0	No stress markers	>1 year old (1–20 years)	Late death: Adult older than 35 years
Yes (1)	Non-breastfed: δ^15^N around or below the female mean value and/or Early weaning-age: < 2 years old		Presence of CO, PH, PR, PL, LEH and/or number of markers ≥ 1	Presence of LEH and/or > LEH Incidence period (years with LEH)	Long bone length below the range of variability and/or HAZ ≤-2 SD	Stature below the group median (M, F) or Z-score < 0	Presence of CO, PH, PR, PL and/or number of markers ≥ 1	≤ 1year old	Early death: Young Adult (20–35 years)

^1^ Lack of breastfeeding or early weaning.

^2^Subadults (non-survivors, individuals who die early in childhood)

^3^Adults (survivors, individuals who survive to later life-stages). CO: cribra orbitalia; PH: porotic hyperostosis; PR: periosteal reactions and other signs of bone infection; PL: periapical lesions; LEH: linear enamel hypoplasia; HAZ (height-for-age Z-score).

## Results

The sample for analyses is composed of 41 individuals, that is, 23 adults (16 males and 8 females, of different age categories) plus 18 juveniles of undetermined sex (S1 File).

### Stature in adults

Stature was estimated in 23 adults (Table 2). The mean height for males is 156.2 cm (SD = 4.73; range: 150.1–166.8; n = 16) and 148.8 cm for females (SD = 5.45; range: 141.1–156.8; n = 7). Males were 9–10 cm taller than females (t = 3.31; df = 21; p = 0.003), with higher heterogeneity in females. Few individuals fit with the strict definition of “short stature” (height ≤ -2SD: 2 females and no males; height ≤ -1SD: 2 females and 4 males).

**Table 2:** Osteobiographic aspects in adults from Jabuticabeira II (n = 23).

N°	Individual	Sex	Age at death	Bone	Length (mm)	Estimated stature (cm)	Z score (according sex)	Age at the end of weaning	# LEH (age-range)	LEH incidence^†^	CO	PH	PR	# PL
1	SEP 34 L2.05	F	YA	femur	361	141.08 ±1.844	-2.738	< 3 yr	2 (4.0–4.8)	0.8	CO	PH	PR	0
				tibia	302									
2	SEP 36A L2.05	M	YA	tibia	327	150.12 ± 2.581	-1.246	~2 yr	6 (2.6–4.9)	2.3	CO	PH	PR	4
3	SEP 37 L2.05	M	OA	radius	236	158.05 ± 3.467	0.434	2–3 yr	6 (2.5–4.8)	2.3	CO	PH		5
4	SEP 40 L2.05	F	Adult	humerus	259	142.94 ± 2.975	-2.396	~2 yr?	7 (2.2–5.6)	3.4	CO	PH	PR	2
5	SEP 41A L2.05	M	Adult	femur	386	150.49 ± 2.627	-1.167	< 3 yr	2 (4.0–4.8)	0.8		PH	PR	3
6	SEP 108 L2.05	F	MA	ulna	239	151.44 ± 3.987	0.837	2–3 yr	no hypoplasia					8
7	SEP 110 prof L2	M	YA	femur	425	159.32 ± 2.289	0.703	< 4 yr	6 (1.5–5.1)	3.6				0
				tibia	352									
8	SEP 11 L1.25	M	MA	femur	403	153.27 ± 2.289	-0.578	2–3 yr	3 (2.4–5.6)	3.2		PH		2
				tibia	334									
9	SEP 12B L1.25	M	YA	humerus	295	154.30 ± 2.975	-0.360	2–3 yr	no hypoplasia					0
10	SEP 24 L1-1.20	M	Adult	ulna	237	151.10 ± 3.615	-1.038	2–3 yr	nr					nr
11	SEP 14 L1.05	F	YA	tibia	338	151.35 ± 1.963	0.853	~2 yr	2 (4.0–4.3)	0.3				0
12	SEP 15A L1.05	M	MA	radius	257	166.83 ± 3.467	2.294	< 3 yr	2 (2.1–4.9)	2.8			PR	11
13	SEP 17 A L1.05	M	MA	femur	430	161.17 ± 2.289	1.095	< 4 yr	no hypoplasia			PH	PR	0
				tibia	359									
14	SEP 41 L1	M	Adult	humerus	290	154.78 ± 3.522	-0.259	2–3 yr	nr					nr
15	SEP 102 L1.75	F	Adult	humerus	284	150.83 ± 2.975	-0.949	2–3 yr	no hypoplasia		CO	PH	PR	0
16	SEP 123 L1.80	M	MA	humerus	299	157.91 ± 3.522	0.405	2–3 yr	nr		nr	nr	PR	nr
17	SEP 107 L1-T1	M	OA	humerus	297	157.22 ± 3.522	0.256	2–3 yr	no hypoplasia		CO	PH		3
18	SEP 2A L6	F	MA	radius	209	147.02 ± 3.012	-0.648	2–3 yr	no hypoplasia		CO	PH		0
19	SEP 114 L6	M	OA	radius	246	162.23 ± 3.467	1.320	2–3 yr	2 (4.4–4.8)	0.8	nr	PH		nr
20	SEP 115B L6	M	MA	femur	398	151.09 ± 2.289	-1.040	~2 yr	2 (4.0–4.8)	0.8		PH		0
				tibia	325									
21	SEP 118 L6	M	YA	femur	402	153.62 ± 2.289	-0.504	2–3 yr	7 (2.9–5.6)	2.7	CO	PH	PR	0
				tibia	337									
22	SEP 121 L6	M	MA	humerus	298	157.56 ± 3.522	0.331	2–3 yr	2 (3.7–4.9)	1.2	CO	PH	PR	8
23	SEP 333A L6	F	YA	radius	233	156.79 ± 3.467	0.145	2–3 yr	nr		nr	nr	PR	nr

* Individuals whose final weaning age was not possible to be estimated, were considered as having been weaned at age 2–3 years. CO: cribra orbitalia; PH: porotic hyperostosis; PR: periosteal reactions and bone infections; #PL: number of periapical lesions of maxilla and mandible. NR: not recordable.

^†^Maximum period between the first and last hypoplasias calculated in years according to Reid and Dean [67].

### Stature in juveniles

Only 18 juveniles from Jabuticabeira II provided adequate preservation for osteometric recording. The estimated height-for-age of each juvenile is show in Table 3. In general, the length of long bones suggests short statures in all juveniles in comparison with reference populations (Table 4). All individuals younger than 1 year of age had femoral bone lengths below or near the 10th percentile of modern USA populations [98]. With five exceptions (individuals 120-L2.05; 10A-L1.25; 115A-L6; 17D2-L1.05; 119A-L6), the femoral lengths of Jabuticabeira II juveniles are closer to the lower limit of Arikara and within the range of Andean individuals (Fig 2).

**Table 3:** Dental age, long bone lengths and estimated stature in juveniles from Jabuticabeira II (n = 18).

N°	Individual	Dental age*		Bone	Length (mm)	Range of long bones lengths (mm)			Estimated stature (cm)		% of adult femur length attained**	Compatible with stunting?
		Ubelaker 1999	Gaither 2004			EUA 20^th^ century^1^	Arikara^2^	Andean^3^	Ruff 2007	Z-score		
1	SEP 35A - L2.05	6±3 m	6±2 m	femur	77	79.4–118.2	62.5–106.0	77.4–131.0	55.93 ± 1.7	M = -6.07	0.19 (0.23)	Yes
										F = -4.89		
2	SEP 17D1—L1.05	6±3 m	6±2 m	humerus	65	79.9–91.6	63.5–89.0	67.0–81.6	?	-	-	Yes
3	SEP 17B- L1.05	6±3 m	6±2 m	humerus	82	79.9–91.6	63.5–89.0	67.0–81.6	?	M = -2.03	-	No
				tibia	83	81.6–98.1	59.5–94.0	65.6–103.0	64.70 ± 1.5	F = -1.10		
4	SEP 41B - L2.05	9±3 m	6±2 m	humerus	72	-	84.0–119.0	70.2–88.9	?	M = -5.56	0.21 (0.26)	Yes
				femur	83	-	92.5–161.0	92.0–110.2	57.75 ± 1.7	F = -6.79		
5	SEP 3F - L6	9±3 m	6±2 m	humerus	75	-	84.0–119.0	70.2–88.9	?	M = -5.66	0.23 (0.26)	Yes
				femur	91	-	92.5–161.0	92.0–110.2	60.33 ± 1.6	F = -4.51		
				tibia	76	-	81.0–131.5	77.4–93.0				
6	SEP 115A-L6	9±3 m	6±2 m	humerus	86	-	84.0–119.0	70.2–88.9	?	M = 0.11	0.27 (0.26)	No
				femur	108	-	92.5–161.0	92.0–110.2	73.52 ± 1.5	F = 0.83		
7	SEP 17D2—L1.05	9±3 m	6±2 m	radius	69	-	84.0–104.0	55.0–72.2	69.33 ± 2.0	M = -1.72	-	No
										F = -0.87		
8	SEP 120—L2.05	12±4 m	9±2 m	humerus	100	97.3–112.1	84.0–119.0	83.8–98.7	?	-	-	No
9	FS30—L1.70	12±4 m	9±2 m	femur	100	128.0–143.0	92.5–161.0	104.5–140.9	62.90 ± 1.7	M = -5.40	0.25 (0.28)	Yes
										F = -4.31		
10	SEP 17D - L1.05	12±4 m	9±2 m	femur	105	128.0–143.0	92.5–161.0	104.5–140.9	64.42 ± 1.7	M = -4.76	0.26 (0.28)	Yes
										F = -3.72		
11	SEP BEBÊ - L1/L2	12±4 m	9±2 m	humerus	96	97.3–112.1	84.0–119.0	83.8–98.7	?	M = -2.98	0.29 (0.28)	No
				femur	116	128.0–143.0	92.5–161.0	104.5–140.9	68.55 ± 1.6	F = -2.08		
				tibia	98	102.6–117.4	81.0–131.5	85.6–101.5				
12	SEP 35B - L2.05	4yr ±12 m	3yr±11m	femur	150	209.7–238.4	208.0–218.0	131.0–180.0	81.71 ± 1.8	M = -5.32	0.37 (0.46)	Yes
				tibia	120	168.5–194.4	165.0–176.0	109.0–139.0		F = -5.04		
13	SEP 101- L2.05	4yr ±12 m	3yr±11m	humerus	121	151.0–171.2	154.0–159.0	107.0–136.5	86.95 ± 2.0	M = -5.05	0.38 (0.46)	Yes
				femur	153	209.7–238.4	208.0–218.0	131.0–180.0	82.84 ± 2.2	F = -4.78		
14	SEP 119A-L6	4yr ±12 m	3yr±11m	humerus	139	151.0–171.2	154.0–159.0	107.0–136.5	94.27 ± 2.0	M = -2.25	0.47 (0.46)	No
				femur	189	209.7–238.4	208.0–218.0	131.0–180.0	94.59 ± 1.8	F = -2.05		
				tibia	160	168.5–194.4	165.0–176.0	109.0–139.0				
15	SEP 16B - L1.05	4yr ±12 m	3yr±11m	humerus	133	151.0–171.2	154.0–159.0	107.0–136.5	91.83 ± 2.0	M = -3.53	0.44(0.46)	Yes
				femur	175	209.7–238.4	208.0–218.0	131.0–180.0	89.21 ± 1.8	F = -3.30		
				tibia	141	168.5–194.4	165.0–176.0	109.0–139.0				
16	SEP 38—L2.05	7yr ±24 m	6yr±20m	humerus	160	190.0–215.5	187.5–204.0	161.0–163.9	104.4 ± 2.8	M = -3.59	0.55 (0.59)	Yes
				femur	220	273.0–308.2	252.0–274.0	183.8–222.0				
									103.08 ± 1.9	F = -3.31		
				tibia	178	215.8–253.8	212.0–229.5	178.0–186.0				
17	SEP 10A- L1.25	7yr ±24 m	6yr±20m	humerus	190	190.0–215.5	187.5–204.0	161.0–163.9	117.75 ± 2.8	M = -1.10	0.65 (0.59)	No
				femur	261	273.0–308.2	252.0–274.0	183.8–222.0	115.87 ± 1.9	F = -0.90		
				tibia	216	215.8–253.8	212.0–229.5	178.0–186.0				
18	SEP 104A - L1.85	15yr±36m	12yr±21m	humerus	250	-	-	237.0–257.0	144.10 ± 3.8	M = -3.67	0.89 (0.97)	Yes
				femur	355	-	345.0–368.0	329.0–338.0				
				tibia	297	-	294.0–319.0	264	140.24 ± 2.9	F = -3.11		

* The dental aging method by Ubelaker [90] was used to classify Arikara [90] and Andean [100] individuals. Dental aging of Gaither [91] figures in this study as reference due to the possibility of precocious dental eruption in our samples.

^1^Maresh [98] included bone lengths between 10th and 90th percentiles (in this case the mean value from males and females). The tables by Maresh do not include an age category for around 9 months, nor data of individuals older than 12 years. In this research, the Maresh’s age cohort of 6m includes individuals between 0–6 months.

^2^Ubelaker [98] includes the complete variability range for each bone

^3^Vega [100] includes the complete variability range for each bone. The Ubelaker’s phase NB-0.5 yrs equals the phases Nac±2m and 6m±3m by Vega [100]; the phase 0.5–1.5 yr equals the phases 9m±3m and 12m±4m. The tables by Vega [100] are applicable without sexing.

** % femur length attained [103] = individual femur length / average femur length of adults (pooled sample). In brackets the expected value calculated from Denver Growth Study data [98].

**Table 4:** Correlations between variables in adults from Jabuticabeira II (females and males pooled).

Z-scores of adult heights (by sex) vs.	n	Spearman's ρ	Sig. (2-tailed)
First LEH	13	-.350	.240
LEH incidence period	13	.223	.464
# LEH	19	-.052	.833
# stress markers in adulthood	23	-.259	.232
In females	7	-.764	**.046**
Age-at-death in adulthood	23	.409	.053
First LEH (adults) vs.			
LEH incidence period	13	-.946	**.001**
in males	10	-.938	**.001**
in males	10	-.979	**.001**
# LEH	13	-.635	**.020**
# stress markers in adulthood	13	-.093	.763
Age-at-death in adulthood	13	-.003	.992
LEH incidence period (adults) vs.			
# LEH	13	.765	**.002**
# stress markers in adulthood	13	.180	.557
Age-at-death in adulthood	13	-.069	.824
# LEH (adults)			
# stress markers in adulthood	19	.367	.122
Age-at-death in adulthood	19	-.231	.341
Age-at-death in adulthood vs.			
# Periapical lesions	23	.493	**.038**
Maximum number of stress markers vs.			
Age-at-death in adulthood	23	.152	.489
Age-at-death in childhood	18	.384	.115
Weaning age vs.			
# stress markers in adulthood	23	-.203	.304
# stress markers in childhood	18	-.617	.140
Age-at-death in adulthood	23	.145	.508
Age-at-death in childhood	18	.529	.222

#: number of. # stress markers in adulthood = maximum number of stress markers present per individual (the sum of CO, HO, LEH, PR and PL).

**Fig 3:**
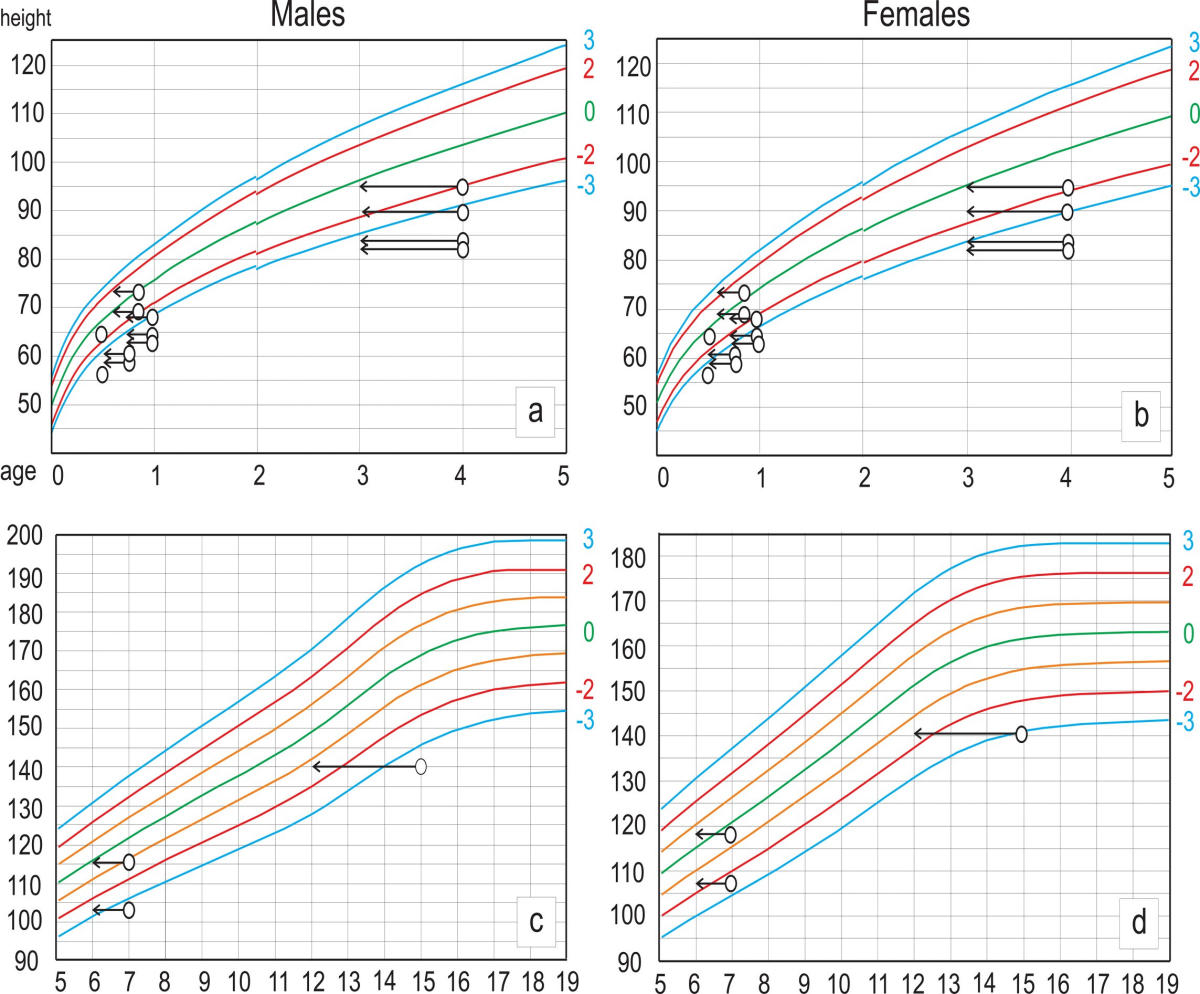
Height-for-age in individuals from Jabuticabeira II in the WHO developmental charts. a) Male individuals aged 0–5 years; b) Female individuals aged 0–5 years; c) Male individuals aged 5–19 years; d) Female individuals aged 5–19 years. Points show height-for-age when age-at-death was estimated according to Ubelaker [90] dental chart. Arrows show height-for-age when age-at-death was estimated using the Gaither [91] chart. Red line: 2σ; Blue line: 3σ.

In juveniles with estimated stature, the Z-scores are well below the mean and lower limits (≤ -2 SD) of contemporary growth curves [50]. That suggests an extremely low height-for-age record in Jabuticabeira II (Fig 3), even when assuming an earlier dental eruption (around one year earlier) following the chart of Gaither [91]. The height estimated from upper limbs is greater than the height estimated from lower limbs in 3 out of 6 evaluated individuals (Table 3). Reduced growth in lower limbs can be interpreted as stunting. Finally, the percentage of adult femur length attained at different ages confirms our observations in most cases.

### Physiological stress markers during childhood and adulthood in Jabuticabeira II

**Fig 4:**
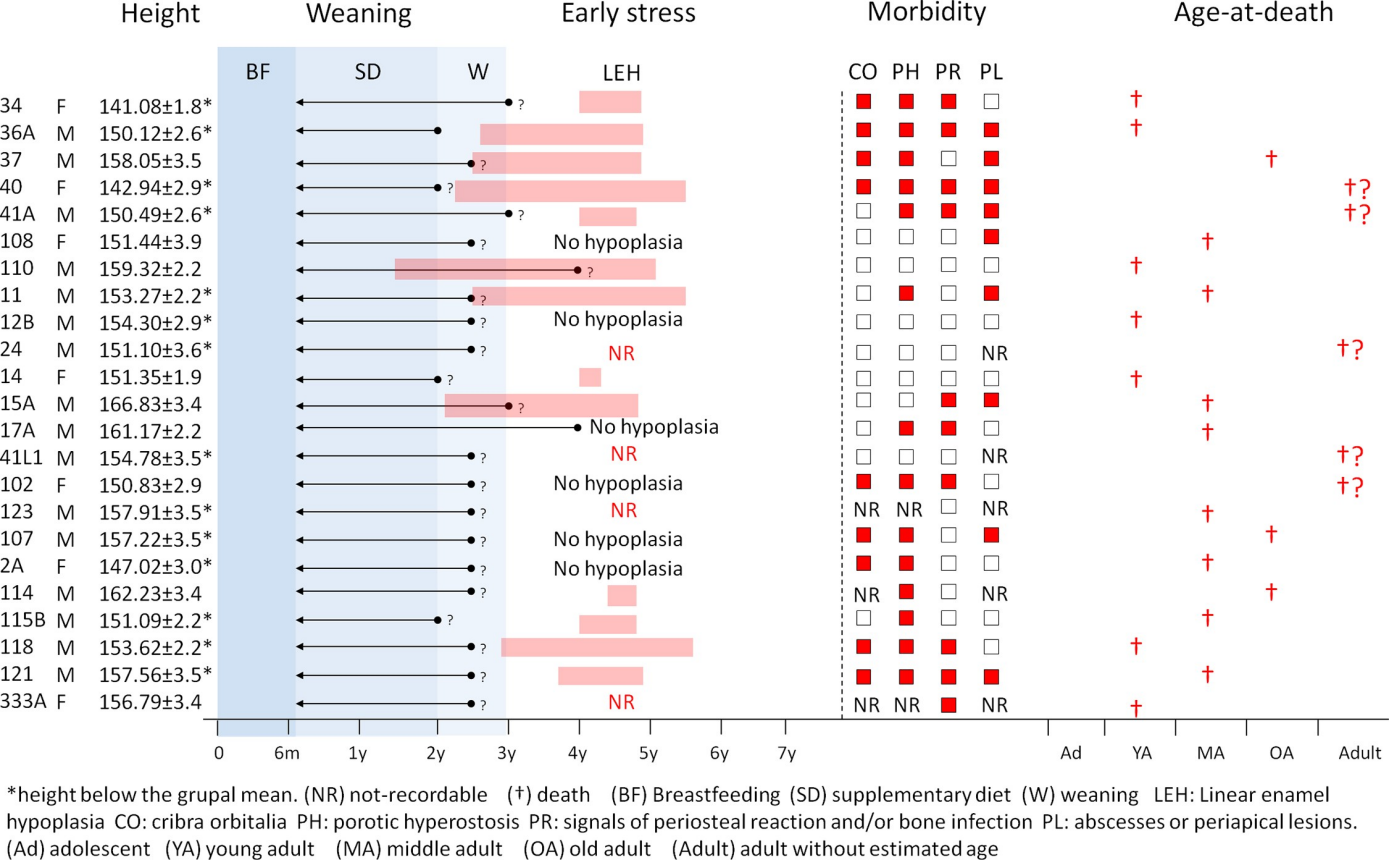
Physiological stress markers per individual in adults from Jabuticabeira II.

The physiological stress markers of the 23 adults assessed are shown in Table 2 and Fig 4. In adults, LEH, which develops during childhood, affected 13 (10/12 males and 3/6 females), among 19 individuals with available dentitions.

When we compare the timing of LEH to individual weaning trajectories (Fig 4), we observe that in Jabuticabeira II, among the 13 adults with LEH, 6 experienced the first LEH a few months before or immediately after the approximate age of the end of weaning, suffering events of physiological stress between 2.1 and 5.6 years of age. Other 6 individuals had the first LEH well after the end of weaning (with physiological stress events between 3.7 and 4.9 years old). Finally, only one individual suffered the first LEH more than one year before weaning (with physiological stress events from 1.5 to 5.1 years of age). Six individuals did not suffer LEH and 4 were not evaluated due to the absence of teeth.

The mean ages at the first and last LEH, despite their inter-individual variability, suggest that, in Jabuticabeira II, the individuals suffered metabolic stress after weaning (mean weaning age = 2.3 years; mean age of the first LEH = 3.02 years, SD = 0.83, n = 17; mean age of the last LEH = 4.90 years, SD = 0.52, n = 17). The time of exposure to etiological factors in individuals with LEH is variable (ranging between 0.3 and 3.6 years of age; mean = 1.9 years; SD = 1.10, n = 17), without differences between sexes (S2 File).

The statistically significant correlations between the age at the first LEH, “LEH incidence period” and the number of LEH (Table 4) suggest that individuals who start earlier with LEH after weaning suffered longer LEH incidence periods and had more LEH events in absolute numbers. All these significant correlations can be interpreted as long periods of recurrent stress during childhood.

The age-at-death in adults (the mean age of each age-range) does not correlate with early stress (age of weaning ρ = -0.145, p = 0.508; duration of the “period of LEH incidence” ρ = -0.069, p = 0.824; number of LEH ρ = -0.231, p = 0.341). Instead, it is possibly more related to acute or chronic aggressions experienced later in life, as the positive correlation between age-at-death and PL suggest (ρ = 0.493, p = 0.038).

**Fig 5:**
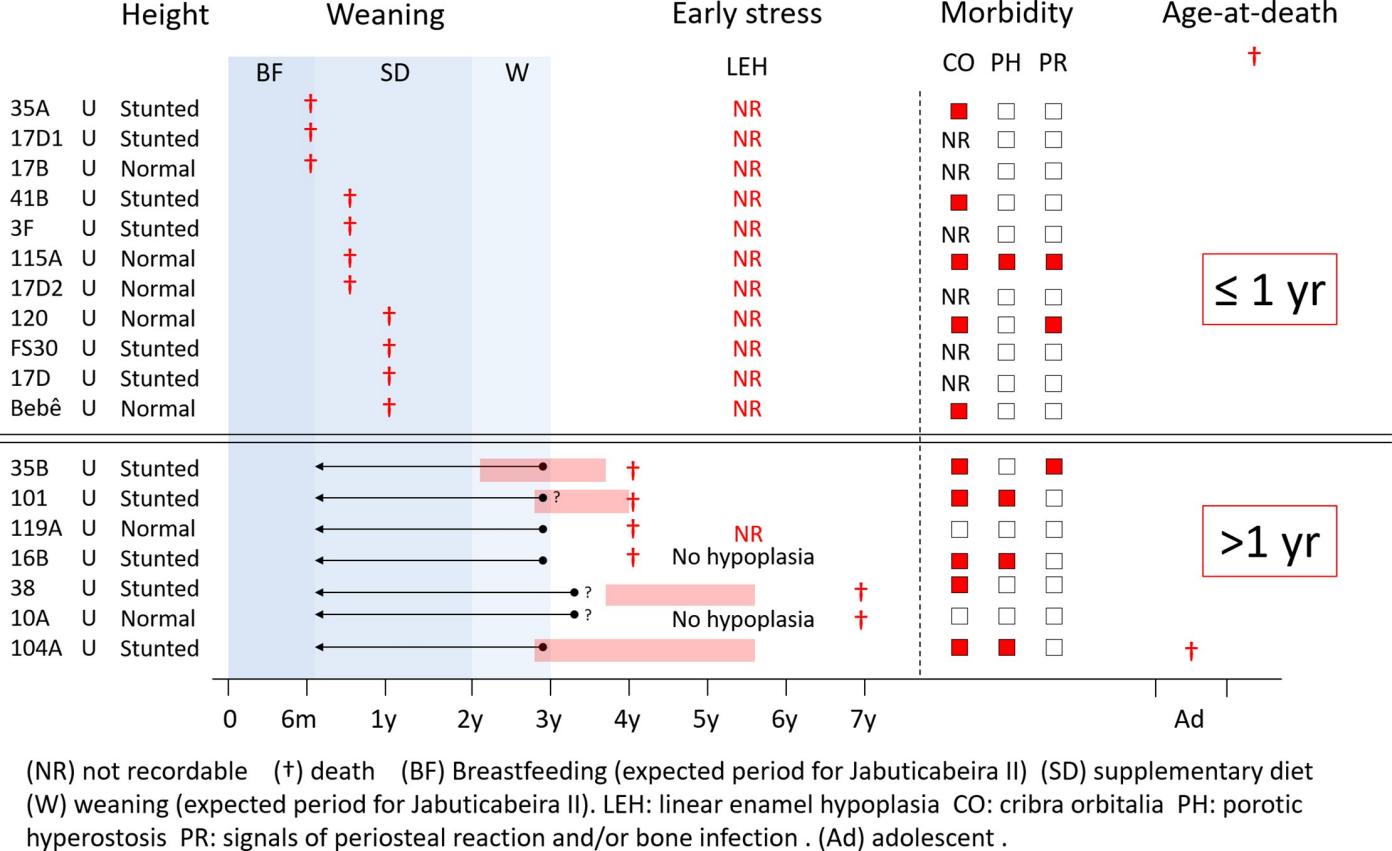
Physiological stress markers by individual in juveniles from Jabuticabeira II.

Only 6 juveniles were available for enamel LEH analysis (those with mixed dentition or exposed teeth). As observed in adults, in all these juveniles LEH appears during weaning periods and lasts several months after the end of weaning (Fig 5).

Among 18 juveniles examined in Jabuticabeira II (Table 5), 10 presented CO (not-recordable = 6) and 4 show PH (not-recordable = 0). All individuals who died before 1 year old showed presence of CO (5/5 with available orbits). Among the 18 juveniles, 7 died after age 1 year and 5 out of these 7 show CO and PH. However, there was no significant difference between ages-at-death <1 year and at > 1 year (CO: *X*^*2*^ = 1.397 p = 0.237; PH: *X*^*2*^ = 2.822 p = 0.092). PR and other signs of infection occurred in 3/18 individuals (not recordable = 0). Only two out of 18 juveniles showed periostitis in more than one bone (died at age 1 and 4 years, respectively) and both cases are consistent with subacute inflammatory processes.

**Table 5:** Osteobiographic aspects in juveniles from Jabuticabeira II (n = 18).

N°	Individual	Dental age*		Compatible with Stunting?	Subadult diet	Age at the end of weaning	# LEH (age range)	LEH incidence^†^	CO	PH	PR
		Ubelaker 1999	Gaither 2004								
1	SEP 35A - L2.05	6±3 m	6±2 m	Yes	breastfeeding, supplementary?	not weaned	nr		CO		
2	SEP 17D1—L1.05	6±3 m	6±2 m	Yes	breastfeeding, supplementary?	nd	nr		nr		
3	SEP 17B- L1.05	6±3 m	6±2 m	No	breastfeeding, supplementary?	not weaned	nr		nr		
4	SEP 41B - L2.05	9±3 m	6±2 m	Yes	breastfeeding, supplementary?	weaned?	nr		CO		
5	SEP 3F - L6	9±3 m	6±2 m	Yes	breastfeeding, supplementary?	not weaned	nr		nr		
6	SEP 115A-L6	9±3 m	6±2 m	No	breastfeeding, supplementary?	not weaned	nr		CO	PH	PR
7	SEP 17D2—L1.05	9±3 m	6±2 m	No	breastfeeding, supplementary?	not weaned	nr		nr		
8	SEP 120—L2.05	12±4 m	9±2 m	No	breastfeeding, supplementary?	not weaned	nr		CO		PR
9	SEP FS30—L1.70	12±4 m	9±2 m	Yes	breastfeeding, supplementary?	nd	nr		nr		
10	SEP 17D - L1.05	12±4 m	9±2 m	Yes	breastfeeding, supplementary?	not weaned	nr		nr		
11	SEP BEBÊ - L1/L2	12±4 m	9±2 m	No	breastfeeding, supplementary?	not weaned	nr		CO		
12	SEP 35B - L2.05	4yr±12 m	3yr±11m	Yes	marine	~3yr	2 (2.2–3.7)	1.5	CO		PR
13	SEP 101- L2.05	4yr±12 m	3yr±11m	Yes	terrestrial	~3yr	3 (2.8–4.0)	2.1	CO	PH	
14	SEP 119A-L6	4yr±12 m	3yr±11m	No	mixed	~3yr	nr				
15	SEP 16B - L1.05	4yr±12 m	3yr±11m	Yes	mixed	< 3yr	no hypoplasia		CO	PH	
16	SEP 38—L2.05	7yr±24 m	6yr±20m	Yes	mixed	> 3yr?	2 (3.7–5.6)	0.5	CO		
17	SEP 10A- L1.25	7yr±24 m	6yr±20m	No	mixed	> 3yr?	no hypoplasia				
18	SEP 104A - L1.85	15yr±36m	12yr±21m	Yes	mixed	< 3yr	6 (2.8–5.6)		CO	PH	

*ND: individuals without isotopic data for weaning. NR: not recordable or teeth not-erupted. Mixed diet means a different diet from the marine diet typical for Jabuticabeira II adults. CO: cribra orbitalia; PH: porotic hyperostosis; PR: periosteal reactions and bone infections.

^†^Maximum number of years between the first and last hypoplasia events according to Reid and Dean 2006 [67].

Among the 23 adults, 9 individuals showed CO (5/16 males; 4/7 females; not-recordable = 3) and 14 showed PH (10/16 males and 4/7 females; not-recordable = 2). PR were observed in 11 individuals (7/16 males and 4/7 females; not recordable = 0). The most frequent lesions found were periostitis in long bones, but there are three cases of osteomyelitis with fistulae or *cloacae* (two in a femur and one in a tibia). Abscesses and other maxillofacial infectious lesions (regardless of their etiology) occurred in 9 adults (7/12 males and 2/6 females; not recordable = 5).

There is a significantly higher proportion of juveniles (10/12) than adults (9/20) affected by CO (*X*^*2*^ = 4.569, p = 0.032) in our sample. Curiously, PH was significantly more prevalent (*X*^*2*^ = 7.704, p = 0.005) among adults (14/21) than juveniles (4/18).

Adults (14/21) also show significantly higher proportions (*X*^*2*^ = 9.854 p = 0.001) of PR than juveniles (3/18). This can be related to the cumulative effect of recurrent metabolic insults during adulthood, but also with more severe or chronic morbidity processes in juveniles that did not allow the development of PR [7,11,14].

The results from adults in Jabuticabeira II are not entirely compatible with the statement that individuals exposed to early-life stress and early weaning age present higher morbidity and lower survival expectations. The Spearman's coefficient did not detect significant correlations between stress markers in any of the other comparative scenarios. Although this could be the result of a sample size bias, it also agrees with the non-specific etiology of stress markers outlined by other bioarchaeological studies [40,105].

Logistic Regression models performed in adults and juveniles were unable to demonstrate predictive relationships between variables. The only significant model (S3 File) shows that adult short height is a significant predictor of age-at-death < 35 years (*X*^2^ = 6.964; p = 0.008; R^2^ Nagelkerke = 0.350; OR = 14.400; CI (95%): 1.375–150.808).

Morbidity processes (CO, HP, PR and infectious diseases markers) in adults -“survivors”- are frequent and could, in theory, be associated with the causes of death. The association, however, is not so clear: among those who exhibited two or more stress markers (13/23 individuals), three deaths occurred before age 35, and seven after age 35, while in the remaining three cases it was not possible to define age of death.

Nevertheless, there is a significant negative correlation between adult height and adult morbidity (the total number of stress markers) in females (ρ = -0.764 p = 0.046), which supports the idea of higher frailty in short height females (Table 5). This could be explained by sex-differences in the distribution of labor (as suggested by activity markers) less access to protein by women than men, among other unknown possible causes.

## Discussion

Small data sets like the one used herein (41 juveniles and adults from the *sambaqui* Jabuticabeira II) may constrain data interpretation, and therefore undermine straightforward conclusions. However, they do not preclude valuable knowledge if used appropriately. The strength of the approach used in the present study lies in the detailed osteobiographic integration of multiple lines of evidence. However, because the sample is not large and complete enough to support extensive generalizations, our interpretations should be considered with caution for extrapolations.

In this study we aim to identify periods of higher vulnerability to health hazards and causes of differential frailty among fisher-hunter-gatherers from Jabuticabeira II. The results indicate that: 1) Jabuticabeira II adults are, in general, of relative short stature; 2) most of the juveniles show statures suggestive of stunting; 3) early stress (LEH in adults and juveniles) is mainly associated with the end of the weaning process or later childhood stages; 4) non-specific physiological stress markers affect high proportion of juveniles and adults suggesting recurrent periods of stress along lifetime; 5) a high proportion of individuals who died during the juvenile period (< 20 years) show cribra orbitalia and porotic hyperostosis; 6) in adults shorter stature is correlated with higher morbidity; and finally, 7) shorter adult stature is a significant predictor of early age-at-death.

### “Stunting” and early stress

Establishing causes for the “short stature” observed in adults from Jabuticabeira II is difficult because many South American indigenous groups (ethnographical and archeological) also show short stature and a high prevalence of stunting [49,56,100]. Several ecological-adaptive hypotheses (and several selective factors) have been postulated to explain the small body size observed in these groups [106–108]. Considering the high heritability of body height, only future paleogenetic research can clarify if the short stature observed was the result of population wide stunting events or the outcome of genetic traits. However, our results suggest, given the high prevalence of bone stress markers, that non-genetic factors such as malnutrition and poor health conditions played an important role in the stature expression in Jabuticabeira II.

The juveniles of Jabuticabeira II show evidence consistent with stunting (short bone length, lower limbs shorter than upper limbs, and short stature), always associated with physiological stress. When we compare the lengths of the long bones of juveniles from Jabuticabeira II to those of modern affluent populations, all individuals, with no exception, are either shorter or at the lower end of the range of variability. When compared to Arikara and Andean individuals, our individuals cluster mainly at the lower limit of the range.

In the Jabuticabeira II individuals who died within their first year of life, height (and possibly also weight) is variable, but always well below the median of the WHO reference curve (HAZ ≤ -2). This growth delay lasts until at least 3–4 years of age, the most sensitive period of growth, when it is more difficult to recover the normal growth trajectory by catch-up growth [49,56]. Some individuals died with more than one year of age, showing Z-scores inside the expected range, could be the evidence of catch-up growth processes [56]. If stunting occurs up to 4 years or later, which seems to be the case in most individuals from Jabuticabeira II, there was no possibility of catch up growth and short adult stature would be an expected consequence.

However, some issues need to be raised. With or without environmental influences, there will be variation in child growth when plotted by biological age, mainly in individuals below one year of age because of the high developmental variability within this age group. Reliable methods to estimate age at death in this age-range are based on histological approaches [109] that we did not use in this research, and caution is necessary when comparing archaeological groups with data retrieved from modern populations [110]. Underestimated stature in all age groups, especially in lower limbs, due to constraints of the methods is also possible [102].

On the other hand, the length of long bones from Jabuticabeira II juveniles, which are shorter in comparison to reference populations, could also indicate precocious dental development. Studies by Gaither [91] and Vega [100] have shown that the Ubelaker method [90] underestimates dental age of South American Indians (which would present an earlier than predicted dental eruption). Despite those considerations, the integration of so many different lines of evidence leads to claim that most Jabuticabeira II juveniles analyzed herein show statures compatible with stunting.

What are the possible causes of the high proportion of deaths in stunted juveniles? There are several factors related to stunting that could be among direct and indirect causes of death.

The number of juveniles with HAZ ≤ 2SD in our sample is 72.7% (13/18). In most of the reported ethnographic groups of Amazonian fisher-hunter-gatherers and horticulturists analyzed with the same WHO parameters the frequencies of diagnosed stunted children (≤ -2SD) range from 20 to ~80% [49,56]. In all those extant populations, with either more or less insertion to market systems, the HAZ mean was invariably negative in comparison to the reference standards [49, 56, 109], and lower prevalence of stunting was associated with greater protein intake [111]. In contrast, the available archaeological evidence does not support lack of protein in Jabuticabeira II [25, 27]. This could indicate a genetic background for shorter statures as possible cause. However, other markers suggest different explanations.

In Jabuticabeira II, the high prevalence of CO and PH (anemia-related markers), in children who died before 1 year of age and were very small compared to their counterparts from reference populations, suggest shortcomings in child feeding and parental investment strategies, as well as increased exposure to environmental threats [112]. On the other hand, since a proportion of all humans will die during the perinatal interval [53], the presence of bone response to environmental challenges and delayed death in Jabuticabeira II children >1 year old could be interpreted as a social ability to keep newborns alive despite stressors.

Adolescent or extremely malnourished pregnant women can generate low birth weight in infants, who then show an increased risk of neonatal, perinatal or infant morbidity and mortality [40, 113]. Although mothers with nutritional deficiencies could transmit such depletions during breastfeeding [72], in Jabuticabeira II these cases appear to be associated with extrinsic factors and not to breastfeeding restrictions. Despite an expected intra-population variation of isotope values, individuals ≤ 1 year old revealed δ^15^N isotopic values ​​compatible with breastfeeding (up to 2σ above the mean values ​​of the female population: [87]). However, recent publications show that high δ^15^N values from collagen (particularly from dentine) do not necessarily reflect breastfeeding and may reflect maternal/infant stress [114–117]. Although occur more frequently in children rather than adults [54], the presence of CO in most of individuals ≤ 1 year suggests a possible association between juvenile mortality and anemia-producing factors (or stress of any etiology) and makes this hypothesis also plausible. Our results do not suggest a unique etiology for CO and PH. Although both conditions appear in juveniles and adults, CO is highly age-specific for young children [54:41,73]. The hypothesis that CO is an early expression of the process that produces HP is disputable in Jabuticabeira II. Several cases of CO are not concomitant with HP. According to Rivera and Lahr [75:92], associations between CO and PH are likely related to anemias such as iron-deficiency, hereditary or some acquired anemias, whereas CO only is related with anemia of chronic disease, anemia of chronic renal failure (a very disputable etiology in this case), or scurvy.

Considering the proximity to the Atlantic rainforest ecosystem, an environment rich in fruiting trees, and anthracological evidence in Jabuticabeira II of many botanical species that provide edible fruits rich in vitamin C [30:156], the diagnosis of scurvy seems unlike. Thus, the most plausible etiology is anemia of chronic disease, related to bacterial, viral and parasitic infections. The proposed etiology for PH, which appears mainly in adults, is anemia related with iron deficiency or megaloblastic anemia produced by vitamin B12 and folic acid depletions linked to chronic diarrheic diseases [72,75]. This etiology seems also plausible for the adults of Jabuticabeira II.

The introduction of a supplementary diet based on marine resources [87] should not be underestimated as a modulating factor for child morbidity-mortality. A marine diet could have represented important risks in terms of parasitic and bacterial infection [118–121], especially in pre-ceramic times. In addition, archaeological evidence suggests that *sambaqui* populations were able to exploit their ecosystem with remarkable efficacy, allowing the establishment of dense sedentary settlements [18,21]. Deficiencies in sanitation, agglomeration of people and consumption of contaminated drinking water therefore cannot be ruled out as explanations for stunting, anemia-related markers or premature death. That is due to the association of all these factors with chronic or recurrent gastrointestinal infections [47,51,121].

In Jabuticabeira II, the introduction of infectious agents along with the supplementary diet seems to have had an immediate lethal effect in some individuals [63]. Considering an expected delay between the introduction of infected foods and the period of effective deterioration of health, the age pattern of infant mortality approximately coincides with the introduction of the supplementary diet (between 4 and 6 months) and the adult diet (after 2–3 years), as estimated by isotopic analyses [87]. A slow height growth throughout the first 3 years of life can also be attributed to diets with low energetic quality, regardless of enough protein intakes [122].

Supporting these inferences, LEH observed in adults and juveniles indicate that children of both sexes were exposed to non-specific physiological stress factors between 1.5 and 5.6 years of age, more severely between 2.5 and 4.8 years. Although is complex correctly correlate age at death in juveniles (characterized by a 6 to 12 months of error) with the onset/end of a disruption of ameloblasts activity that is visible only after enamel maturation [67–69], a clear trend is noticeable. In the cases examined in this study, LEH either begins a few months around the end of weaning (10/17) or much later (7/17).

Considering that hypoplasias are more strongly associated with acute systemic infections (with fever episodes [123:186;^124^]) than with malnutrition [125], those physiological stressors possibly were infectious diseases such as diarrhea, gastrointestinal parasites, respiratory infections, and non-specific infant infections), maybe linked to the introduction of adult diet, poor hygiene and fatal outcomes [40,51]. Thus, although a link between morbidity and mortality is possible, the definitive causes of death in these children remain obscure, as in most archeological human remains.

Evidence of a relation between infant malnutrition and LEH coincident with weaning patterns has been reported for contemporary populations [71,126]. However, high frequencies of LEH have also been reported for post-weaning ages in historical populations, suggesting that hypoplasia reflect stress produced by the introduction of the adult diet or other nutritional threats [40, 72, 127].

In summary, apart from some reservations about the accuracy of isotopically-determined weaning ages and other potential confounding factors related with inter-individual variability and the constrains of the methods used for age estimations [69, 100, 117], our results show shared patterns among several individuals.

### The “intermittent stress of low lethality” model

Our data suggest that the juveniles from Jabuticabeira II would have experienced recurrent periods of stress (possibly in the form of infectious, diarrheal and parasitic diseases) associated with the introduction of a “high risk” diet (possibly contaminated marine food in form of supplementary and adult diets). LEH recorded in adults indicate that the individual survived early-life insults, whereas in juveniles LEH suggest an association with (unknown) cause of death. In Jabuticabeira II, the “survivors” would have lived a life of recurrent periods of morbidity (anemia, infections, and non-specific stress), most likely associated with the parasitic burden of the ecosystem and limited sanitation as the main etiological factors, and less probably due to malnutrition.

It has been hypothesized that individuals with more stress episodes during childhood are more prone to higher morbidity and early mortality [4,10,11,13], in agreement with the “plasticity/constrain” model [4,15]. According to Temple [4], this model would be confirmed by a negative correlation between the age at the first LEH and the number of LEH episodes, and by a positive correlation between the age at the first LEH and age-at-death. In Jabuticabeira II the influence of early stress in adult mortality has not been categorically proven and only the first condition (recurrent early stress) was confirmed.

According to the Osteological Paradox, a positive correlation between high prevalence of bone lesions and increased age would reflect increased resilience and low underlying relative frailty [7,128]. CO and PH are most likely developed in the first few years of life, but their presence in adulthood (affecting between 70–90% of the entire population of Jabuticabeira II: [104]), as active or healed lesions, suggest incidence of metabolic stress possibly related to anemia.

In Jabuticabeira II, the only statistically significant correlation was detected between periapical lesions and age-at-death. This suggests more frequent lesions in groups of older individuals, which is normally associated with less frailty [7]. However, pericapical lesions could be also interpreted as age-related effect of poor oral health, linked to several idiosyncratic and/or local conditions (carious lesions, severe dental wear, osteoporosis, underlying disease, etc. [32,85,86]). This pattern was associated with more frailty in older ages in medieval dense populations [129].

Based on our skeletal analyses, it is impossible to know if PR and infections were recurrent or a one-time event, but some of them (multifocal periosteal reactions related with specific infections such as treponematoses: [36]) suggest “chronicity” or “non-lethality” [7]. The cumulative effect of these metabolic insults could be related to the premature or delayed death of adults. However, we could also not categorically show this in our sample.

Adult mortality would also be modulated by life-threatening acute infections (for those adults who exhibit osteomyelitis or even some potentially fatal dental abscesses), and other imponderable causes, which are not necessarily traceable when analyzing bones. Infection could have happened at any point in individual’s life.

Because we could not categorically prove the relation between early stress and premature death, and higher morbidity (the sum of stress indicators in individuals) is apparently not related to younger ages-at-death, the data obtained in this study are not entirely consistent either with the “plasticity/constrain” model nor the “predictive adaptive response” model [4,15,17]. Instead our data best fits to a model of “intermittent stress of low lethality”: the adaptation to recurrent metabolic insults of low impact that allows survival to reproductive age with relative success, overcoming environmental aggressions in physiological, physical and cognitive terms. This “intermittent stress of low lethality” is the new model we propose as working hypothesis to be tested in populations similar to Jabuticabeira II.

Jabuticabeira II individuals survive significant growth disorders, recurrent juvenile stressors (possibly anemia-related factors more likely associated with infections linked to high parasitic loads rather than malnutrition), and repetitive infections along their adult life, but not necessarily die at earlier ages.

The adaptation to recurrent stressors of relative low lethality imposed by the environment, although generous, does not disagree with previous studies that conclude that *sambaqui* were constructed and inhabited by flexible, successful, stable and growing populations [18, 27]. The high amount of available protein, a stable environment and/or other unknown cultural buffers could explain their relative individual success and the enormous longevity of their culture.

## Supporting information

S1 File (DOCX)

S2 File (DOCX)

S3 File (DOCX)

## References

[1] Pennington RL. Causes of early human population growth. Am J Phys Anthropol.1996; 99: 259–274. 10.1002/(SICI)1096-8644(199602)99:2<259::AID-AJPA4>3.0.CO;2-U 8967327

[2] Sellen DW, Smay DB. Relationship between subsistence and age at weaning in “preindustrial” societies. Hum Nat. 2001; 12: 47–87. 10.1007/s12110-001-1013-y 26191819

[3] Barker DJP. Developmental Origins of Chronic Disease. Public Health. 2012;126: 185–189. 10.1016/j.puhe.2011.11.014 22325676

[4] Temple DH. Plasticity and Constraint in Response to Early-life Stressors among Late/Final Jomon Period Foragers from Japan: Evidence for Life History Tradeoffs from Incremental Microstructures of Enamel. Am J Phys Anthropol. 2014; 155: 537–545. 10.1002/ajpa.22606 25156299

[5] Cohen MN, Crane-Kramer GM. (editors). Ancient health: Skeletal indicators of agricultural and economic intensification Gainesville: University Press of Florida; 2008.

[6] Sparacello VS, Vercellotti G, d’ Ercole V, Coppa A. Social reorganization and biological change: An examination of stature variation among Iron Age Samnites from Abruzzo, central Italy. Int J Paleopathol. 2017; 18: 9–20. 10.1016/j.ijpp.2017.07.003 28888397

[7] Wood JW, Milner GR, Harpending HC, Weiss KM. The osteological paradox: Problems of inferring prehistoric health from skeletal samples. Curr Anthropol. 1992; 33: 343–370.

[8] Wright L, Yoder CJ. Recent Progress in Bioarchaeology: Approaches to the Osteological Paradox. J Archaeol Res. 2003;11(1): 43–70.

[9] DeWitte SN, Stojanowski CM. The osteological paradox 20 years later: past perspectives, future directions. J Archeol Res. 2015; 234: 397–450.

[10] Armelagos GJ, Goodman AH, Harper KN, Blakey ML. Enamel hypoplasia and early mortality: bioarcheological support for the Barker hypothesis. Evol Anthropol. 2009; 18: 261–271.

[11] DeWitte SN. Differential Survival Among Individuals with Active and Healed Periosteal New Bone Formation. Int J Paleopathol. 2014; 7: 38–44. 10.1016/j.ijpp.2014.06.001 29539489

[12] Temple DH, Goodman AH. Bioarchaeology has a “health” problem: conceptualizing “stress” and “health” in bioarchaeological research. Am J Phys Anthropol. 2014; 155: 186–191. 10.1002/ajpa.22602 25137442

[13] Yaussy SL, DeWitte SN, Redfern R. Frailty and famine: patterns of mortality and physiological stress among victims of famine in medieval London. Am J Phys Anthropol. 2016;160: 272–283. 10.1002/ajpa.22954 26854255

[14] Lewis ME. The Paleopathology of Children. Cambridge: Cambridge University Press; 2018.

[15] Worthman CM, Kuzara J. Life history and the early origins of health differentials. Am J Hum Biol. 2005;17: 95–112. 10.1002/ajhb.20096 15611966

[16] Daniel H Temple,. Bioarchaeological evidence for adaptive plasticity and constraint: Exploring life‐history trade‐offs in the human past. Evol Anthropol. 2019; 28:34–46. 10.1002/evan.21754 30561095

[17] Gluckman PD, Hanson MA, Beedle AS. Early life events and their consequences for later disease: a life history and evolutionary perspective. Am J Hum Biol. 2007;19: 1–19. 10.1002/ajhb.20590 17160980

[18] Lima TA. Em busca dos frutos do mar: os pescadores-coletores do litoral centro-sul do Brasil. Revista da USP. 1999–2000; 44: 270–327. 10.11606/issn.2316-9036.v0i44p270-327

[19] Okumura MMM, Eggers S. The People of Jabuticabeira II: Reconstruction of the Way of Life in a Brazilian Shellmound. Homo. 2005; 55: 263–281. 10.1016/j.jchb.2004.10.001 15803771

[20] Mendonça de Souza SMF, Wesolowski V, Rodrigues-Carvalho C. Teeth, nutrition, anemia, infection, mortality: costs of lifestyle at the coastal Brazilian sambaquis In: Humans: Evolution and Environment. vol. 22 BAR International Series 2026; 2009 pp. 33–40.

[21] DeBlasis P, Kneip A, Scheel-Ybert R, Giannini P, Gaspar MD. Sambaquis e Paisagem: Dinâmica Natural e Arqueologia Regional no Litoral Sul do Brasil. Arqueología Suramericana / Arqueologia Sul-americana. 2007; 31: 29–61. Available from: https://leiaufsc.files.wordpress.com/2013/03/5-1-deblasis-p-kneip-a-scheel-ybert-r-giannini-p-c-gaspar-m-d-sambaquis-e-paisagem.pdf.

[22] Bendazolli CS. O processo de formação dos sambaquis: uma leitura estratigráfica do sítio Jabuticabeira II SC. M.Sc. Thesis, Museu de Arqueologia e Etnologia da Universidade de São Paulo, São Paulo. 2007. Available from: https://teses.usp.br/teses/disponiveis/71/71131/tde-04072007-152907/en.php

[23] Giannini PCF, Villagrán XS, Fornari M, Do Nascimento DRJ, Menezes PML, Tanaka APB, et al Interações entre evolução sedimentar e ocupação humana pré-histórica na costa centro-sul de Santa Catarina, Brasil. Bol. Mus. Para. Emílio Goeldi. Cienc. Hum. 2010; 5 (1):105–128. 10.1590/S1981-81222010000100008

[24] INMET Instituto Nacional de Meteorologia Série Histórica, Dados diários-Laguna SC; 2015. Consulted in September 2016. Available from: http://www.inmet.gov.br/portal/index.php?r=bdmep/bdmep

[25] Klökler DM. Food for Body and Soul: mortuary ritual in shell mounds Laguna-Brazil. Ph.D. Dissertation, University of Arizona, Tucson. 2008. Available from: https://repository.arizona.edu/handle/10150/193697

[26] Kneip A, Farias D, DeBlasis P. Longa duração e territorialidade da ocupação sambaquieira na laguna de Santa Marta, Santa Catarina. Revista de Arqueologia. 2018; 31(1): 25–51. Available from: https://www.researchgate.net/publication/326075716_Longa_duracao_e_territorialidade_da_ocupacao_sambaquieira_na_laguna_de_Santa_Marta_Santa_Catarina

[27] Colonese AC, Collins M, Lucquin A, Eustace M, Hancock Y, Ponzoni RAR, et al Long-term resilience of Late Holocene coastal subsistence system in southeastern South America. PLoS ONE. 2014; 9 (4), e93854: 1–13. 10.1371/journal.pone.0093854 24718458 PMC3981759

[28] Abbas AR. Os sepultamentos de Jabuticabeira II, SC. Insights e inferências sobre padrões fenotípicos, análise de modo de vida e organização social através de marcadores de estresse músculo-esqueletal. M.Sc. Thesis, Instituto de Biociências, Universidade de São Paulo. 2013. Available from: https://teses.usp.br/teses/disponiveis/41/41131/tde-24072013-084501/pt-br.php

[29] Petronilho CC. Comprometimento Articular como um marcador de atividades em um grande sambaqui-cemitério. M.Sc. Thesis. Universidade de São Paulo, Instituto de Biociências, São Paulo. 2005. Available from: https://bdpi.usp.br/item/001480821

[30] Bianchini GF. Fogo e paisagem: evidências de práticas rituais e construção do ambiente a partir da análise antracológica de um sambaqui no litoral sul de Santa Catarina. M.Sc. Thesis, Museu Nacional da Universidade Federal do Rio de Janeiro. 2008. Available from: http://www.museunacional.ufrj.br/arqueologia/index.php/discentes/turma-2006-2-mestrado/248-gina-faraco-bianchini

[31] Boyadjian CHC, Eggers S, Scheel-Ybert R. Evidence of plant foods obtained from the dental calculus of individuals from a Brazilian shell mound In: Hardy K, Kubiak-Martens L, editors. Wild harvest: Plants in the hominine and pre-agrarian human world. Studying Scientific Archaeology. Oxford: Oxbow Books; 2016 pp. 215–240.

[32] Pezo-Lanfranco L. Evidence of variability in carbohydrate consumption in prehistoric fisher-hunter-gatherers of Southeastern Brazil: Spatiotemporal trends of oral health markers. Am J Phys Anthropol. 2018; 167: 507–523. 10.1002/ajpa.23681 30159869

[33] Villagrán XS, Klökler DM. Nishida P, Gaspar MD, DeBlasis P. Lecturas estratigráficas: Arquitectura funeraria y depósito de residuos en el sambaquí Jabuticabeira II. Lat Am Antiq. 2010; 21: 195–227.

[34] Okumura MMM, Eggers S. O que a biologia não explica: grupos de afinidade no sambaqui Jabuticabeira II (Jaguaruna, SC). R Museu Arq Etn. 2012; 22: 97–109.

[35] Storto C, Eggers S, Lahr M. Estudo preliminar das paleopatologias da população do Sambaqui Jabuticabeira II, Jaguaruna, SC. R Museu Arq Etn. 1999; 9: 61–71.

[36] Filippini J, Pezo-Lanfranco L, Eggers S. Systematic Regional Study of Treponematoses in Pre-Columbian Brazilian Shell Mounds (Sambaquis). Chungará 2019; 51 (3): 403–425.

[37] Saul FP, Saul JM. Osteobiography: A Maya example In: Iscan MY, Kennedy KAR, editors. Reconstruction of Life from the Skeleton. New York: Alan R. Liss; 1989 pp. 287–302.

[38] Stodder ALW, Palkovich AM. Osteobiography and Bioarchaeology In: Stodder A, Palkovich AM, editors. The Bioarchaeology of Individuals. Gainesville, FL: University Press of Florida; 2012 pp. 1–10.

[39] Hosek L, Robb J. Osteobiography: A Platform for Bioarchaeological Research. Bioarch Int. 2019; 3 (1): 1–15. 10.5744/bi.2019.1005 PMC7254840 32467863

[40] Geber J. Skeletal manifestations of stress in child victims of the Great Irish Famine 1845–1852: Prevalence of enamel hypoplasia, Harris lines, and growth retardation. Am J Phys Anthropol. 2014; 155:149–161. 10.1002/ajpa.22567 25043577

[41] Walker R, Gurven M, Hill K, Migliano A, Chagnon N, De Souza R, et al Growth Rates and Life Histories in Twenty-Two Small-Scale Societies. Am J Hum Biol. 2006; 18: 295–311. 10.1002/ajhb.20510 16634027

[42] Vercellotti G, Piperata BA, Agnew AM, Wilson WM, Dufour DL, Reina JC, et al Exploring the multidimensionality of stature variation in the past through comparisons of archaeological and living populations. Am J Phys Anthropol. 2014;155: 229–242. 10.1002/ajpa.22552 24894916 PMC7424595

[43] Silventoinen K, Sammalisto S, Perola M, Boomsma DI, Cornes BK, Davis C, et al Heritability of adult body height: a comparative study of twin cohorts in eight countries. Twin Res. 2003; 6: 399–408. 10.1375/136905203770326402 14624724

[44] Lettre G. Genetic regulation of adult stature. Curr Opin Pediatr. 2009; 21: 515–522. 10.1097/MOP.0b013e32832c6dce 19455035

[45] Grunauer M, Jorge AAL. Genetic short stature. Growth Horm IGF Res. 2018; 38: 29–33. 10.1016/j.ghir.2017.12.003 29249624

[46] Jelenkovic A, Sund R, Hur YM, Yokoyama Y, Hjelmborg JV, Moller S, et al Genetic and environmental influences on height from infancy to early adulthood: an individual based pooled analysis of 45 twin cohorts. Sci. Rep. 2016; 6: 28496 10.1038/srep28496 27333805 PMC4917845

[47] Bogin B. Patterns of Human Growth Cambridge Studies in Biological and Evolutionary Anthropology 23. Cambridge: Cambridge University Press;1999.

[48] Mascie-Taylor CGN, Lasker GW. Biosocial correlates of stature in a British national cohort. J Biosoc Sci. 2005; 37 (2): 245–251. 15768777 10.1017/s0021932004006558

[49] Zhang R, Undurraga EA, Zeng W, Reyes-García V, Tanner S, Leonard WR, et al Catch-up growth and growth deficits: Nine-year annual panel child growth for native Amazonians in Bolivia. Ann Hum Biol. 2016; 43: 304–315. 10.1080/03014460.2016.1197312 27251215 PMC5392255

[50] WHO Multicentre Growth Reference Study Group. WHO Child Growth Standards: Methods and development: Length/height-for-age, weight-for-length, weight-for-height and body mass index-for-age: Methods and development. World Health Organization, Geneva. 2006. Available from: http://www.who.int/childgrowth/standards/technical_report/en/index.html

[51] King SE, Ulijaszek SJ. Invisible Insults during growth and development: contemporary theories and past populations In: Hoppa R, Fitzgerald CM. Human Growth in the Past: Studies from Bones and Teeth. Cambridge Studies in Biological and Evolutionary Anthropology. Cambridge: Cambridge University Press; 1999 pp. 161–182.

[52] Bogin B, Smith P, Orden AB, Varela Silva MI, Loucky J. Rapid change in height and body proportions of Maya American children. Am J Hum Biol. 2002; 14: 753–761. 10.1002/ajhb.10092 12400036

[53] Lewis ME. The Bioarchaeology of Children: Perspectives from biological and forensic anthropology. Cambridge: Cambridge University Press; 2007.

[54] Larsen CS. Bioarchaeology: Interpreting behavior from the human skeleton 2^nd^ ed. Cambridge: Cambridge University Press; 2015.

[55] Martorell R. Interrelationships between diet, infectious diseases and nutritional status In: Greene L, Johnston FE, editors. Social and the biological predictors of nutritional status, physical growth and neurological development. New York: Academic Press; 1980 pp.81–106.

[56] Ferreira AA, Welch JR, Cunha GM, Coimbra CEA. Physical growth curves of indigenous Xavante children in Central Brazil: results from a longitudinal study 2009–2012. Ann Hum Biol. 2016; 43 (4): 293–303. 10.1080/03014460.2016.1195445 27239686

[57] Martorell R, Kettel Khan L, Schroeder DG. Reversibility of stunting: Epidemiological findings in children from developing countries. Eur J Clin Nutr. 1994; 48 Suppl 1: S45–S57. 8005090

[58] Paajanen TA, Oksala NKJ, Kuukasjarvi P, Karhunen PJ. Short stature is associated with coronary heart disease: a systematic review of the literature and a meta-analysis. Eur Heart J. 2010; 31:1802–1809. 10.1093/eurheartj/ehq155 20530501

[59] Almond D, Currie J. Killing Me Softly: The Fetal Origins Hypothesis. J Econ Perspect. 2011; 253: 153–172. 10.1257/jep.25.3.153 25152565 PMC4140221

[60] Lönnerdal B. Breast milk: A truly functional food. Nutrition. 2000; 16: 509–511. 10.1016/s0899-9007(00)00363-4 10906538

[61] Quigley MA, Kelly YJ, Sacker A. Breastfeeding and hospitalization for diarrheal and respiratory infection in the United Kingdom millennium study. Pediatrics, 2007; 119: e837–e842. 10.1542/peds.2006-2256 17403827

[62] Black RE, Allen LH, Bhutta ZA, Caulfield LE, de Onis M, Ezzati M, et al Maternal and child undernutrition: Global and regional exposures and health consequences. Lancet. 2008; 371: 243–260. 10.1016/S0140-6736(07)61690-0 18207566

[63] Katzenberg MA, Herring DA, Saunders SR. Weaning and infant mortality: evaluating the skeletal evidence. Yearb Phys Anthropol. 1996; 39: 177–199.

[64] Ortner DJ. Identification of Pathological Conditions in Human Skeletal Remains, 2nd ed. New York: Academic Press; 2003.

[65] Hillson S. Tooth Development in Human Evolution and Bioarchaeology. Cambridge: Cambridge University Press; 2014.

[66] Hillson S, Bond S. Relationship of enamel hypoplasia to the pattern of tooth crown growth: a discussion. Am J Phys Anthropol. 1997; 104: 89–103. 10.1002/(SICI)1096-8644(199709)104:1<89::AID-AJPA6>3.0.CO;2-8 9331455

[67] Reid DJ, Dean MC. Variation in modern human enamel formation times. J Hum Evol. 2006; 50: 329e346. 16300817 10.1016/j.jhevol.2005.09.003

[68] Guatelli-Steinberg D. Using perikymata to estimate duration of growth disruption in fossil hominin teeth: issues of methodology and interpretation In: Irish JD, Nelson GC, editors. Technique and Application in Dental Anthropology. Cambridge: Cambridge University Press; 2008 pp. 71–86.

[69] Nava A, Frayer DW, Bondioli L. Longitudinal analysis of the microscopic dental enamel defects of children in the Imperial Roman community of Portus Romae (necropolis of Isola Sacra, 2nd to 4th century CE, Italy). J Archaeol Sci Rep. 2019; 23: 406–415.

[70] Goodman A, Rose J. Dental Enamel Hypoplasias as Indicator of Nutritional Status In: Kelley MA, Larsen CS, editors. Advances in Dental Anthropology. New York: Wiley-Liss;1991 pp. 279–293.

[71] Nelson S, Albert JM, Geng C, Curtan S, Lang K, Miadich S, et al Increased enamel hypoplasia and very low birthweight infants. J Dent Res. 2013; 92:788–794. 10.1177/0022034513497751 23857641 PMC3744269

[72] Sandberg PA, Sponheimer M, Lee-Thorp J, Van Gerven D. Intra-Tooth Stable Isotope Analysis of Dentine: A Step toward Addressing Selective Mortality in the Reconstruction of Life History in the Archaeological Record. Am J Phys Anthropol. 2014; 155: 281–293. 10.1002/ajpa.22600 25156177

[73] Walker PL, Bathurst R, Richman R, Gjerdrum T, Andrushko V. The causes of porotic hyperostosis and cribra orbitalia: A reappraisal of the iron-deficiency anemia hypothesis. Am J Phys Anthropol. 2009; 139: 109–125. 10.1002/ajpa.21031 19280675

[74] Brickley MB. Cribra orbitalia and porotic hyperostosis: A biological approach to diagnosis. Am J Phys Anthropol. 2018; 167: 896–902. 10.1002/ajpa.23701 30259969

[75] Rivera F, Lahr MM. New Evidence Suggesting a Dissociated Etiology for Cribra Orbitalia and Porotic Hyperostosis. Am J Phys Anthropol. 2017; 164:76–96. 10.1002/ajpa.23258 28594081

[76] Roberts CA, Manchester K. The Archaeology of Disease. Ithaca, New York: Cornell University Press; 2005.

[77] Paine RR, Brenton BP. The paleopathology of pellagra: investigating the impact of prehistoric and historical dietary transitions to maize. J Anthropol Sci. 2006; 84: 125–135.

[78] Weston DA. Investigating the specificity of periosteal reactions in pathology museum specimens. Am J Phys Anthropol. 2008; 137: 48–59. 10.1002/ajpa.20839 18398845

[79] Geber J, Murphy E, Scurvy in the Great Irish famine: evidence of vitamin C deficiency from a mid-19th century skeletal population. Am J Phys Anthropol. 2012; 148: 512–524. 10.1002/ajpa.22066 22460661 PMC3467765

[80] Chen EM, Masih S, Chow K, Matcuk G, Patel D. Periosteal reaction: review of various patterns associated with specific pathology. Contemporary Diagnostic Radiology. 2012; 35: 1–6.

[81] Dewitte SN, Bekvalac J. The association between periodontal disease and periosteal lesions in the St. Mary Graces cemetery, London, England A.D. 1350–1538. Am J Phys Anthropol. 2011;146 (4): 609–618. 10.1002/ajpa.21622 21997205

[82] Sheiham A. Claims that periodontal treatment reduces costs of treating five systemic conditions are questionable. J Evid Based Dent Pract. 2015; 15(1): 35–36. 10.1016/j.jebdp.2015.01.001 25666581

[83] Williams RC, Barnett AH, Claffey N. et al The potential impact of periodontal disease on general health: a consensus view. Curr Med Res Opin. 2008; 24: 1635–1643. 10.1185/03007990802131215 18452645

[84] Pizzo G, Guiglia R, Lo Russo L, Campisi G. Dentistry and internal medicine: from the focal infection theory to the periodontal medicine concept. Eur J Intern Med. 2010; 21 (6): 496–502. 10.1016/j.ejim.2010.07.011 21111933

[85] Falcao A, Bullón P. A review of the influence of periodontal treatment in systemic diseases. Periodontal 2000. 2019; 79(1):117–128. 10.1111/prd.12249 30892764

[86] Sapp P, Eversole L, Wysocki GP. Patología oral y maxilofacial contemporánea Madrid: Harcourt Brace de España; 1998.

[87] Pezo-Lanfranco L, DeBlasis P, Eggers S. Weaning process and juvenile diets in a monumental Brazilian shellmound. J Archaeol Sci Rep. 2018; 22: 452–469.

[88] Buikstra J, Ubelaker D. Standards for data collection from human skeletal remains Fayetteville, Arkansas: Arkansas Archeological Survey Research Series # 44; 1994.

[89] Scheuer L, Black S. Developmental Juvenile Osteology. Somerset: Academic Press; 2000.

[90] Ubelaker D. Human Skeletal Remains. Washington: Taraxacum; 1999.

[91] Gaither C. A growth and development study of coastal prehistoric Peruvian population. Ph.D. Dissertation. Graduate School, Tulane University. 2004. Available from: https://digitallibrary.tulane.edu/islandora/object/tulane%3A25578

[92] Ruff C. Body size prediction from juvenile skeletal remains. Am J Phys Anthropol. 2007; 133: 608–716. 10.1002/ajpa.20568 17295297

[93] Giannechini M, Moggi-Cecchi J. Stature in Archeological Samples from Central Italy: Methodological Issues and Diachronic Changes. Am J Phys Anthropol. 2008; 135: 284–292. 10.1002/ajpa.20742 18000888

[94] Pomeroy E, Stock J. Estimation of stature and body mass from the skeleton among coastal and mid-altitude Andean populations. Am J Phys Anthropol. 2012; 147: 264–279. 10.1002/ajpa.21644 22183641

[95] Genovés S. Proportionality of the long bones and their relation to stature among Mesoamericans. Am J Phys Anthropol. 1967; 26: 67–77. 10.1002/ajpa.1330260109 5633729

[96] Del Angel A, Cisneros HB. Technical Note: Modification of Regression Equations Used to Estimate Stature in Mesoamerican Skeletal Remains. Am J Phys Anthropol. 2002;125: 264–265. 10.1002/ajpa.10385 15386254

[97] WHO Multicentre Growth Reference Study Group. WHO Anthro version 3.2.2 and macros. World Health Organization, Geneva. 2011. Available from: http://www.who.int/childgrowth/software/en/

[98] Maresh MM. Measurements from roentgenograms In: McCammon RW, editor. Human Growth and Development. Springfield IL: C.C. Thomas;1970 pp. 157–200.

[99] Schillaci MA, Nikitovic D, Akins NJ, Tripp L, Palkovich AM. Infant and juvenile growth in ancestral Pueblo Indians. Am J Phys Anthropol. 2011; 145: 318–326. 10.1002/ajpa.21509 21469079

[100] Vega MC. Estimación de edad en juveniles: estudio dental y métrico en poblaciones andinas peruanas. M.Sc. Thesis. Pontificia Universidad Católica del Perú, Lima. 2009. Available from: http://tesis.pucp.edu.pe/repositorio/handle/20.500.12404/1531

[101] Buschang PH. Differential long bone growth of children between two months and eleven years of age. Am J Phys Anthropol.1982; 58: 291–295. 10.1002/ajpa.1330580307 7124922

[102] Cardoso HFV. A Test of Three Methods for Estimating Stature from Immature Skeletal Remains Using Long Bone Lengths. J Forensic Sci. 2009; 54 (1): 13–19. 10.1111/j.1556-4029.2008.00916.x 19040671

[103] Humphrey L. Growth studies of past populations: An overview and an example In Cox M, Mays S, editors. Human osteology in archaeology and forensic sciences. London: Greenwich Medical Media; 2000 pp 23–38.

[104] Giusto MND. Os sambaquieiros e os outros: estresse e estilos de vida na perspectiva da longa duração–o caso do Litoral Sul de Santa Catarina. M.Sc. Thesis, Museu de Arqueologia e Etnologia da Universidade de São Paulo, São Paulo. 2017. Available from: https://teses.usp.br/teses/disponiveis/71/71131/tde-15012018-164309/pt-br.php

[105] Mays SA. The relationship between Harris lines and other aspects of skeletal development in adults and juveniles. J Archaeol Sci. 1995; 22: 511–520.

[106] Stinson S. Variation in body size and shape among South American Indians. Am J Hum Biol. 1990; 2: 37–51. 10.1002/ajhb.1310020105 28520262

[107] Schell LM, Knutsen KL Environmental effects on growth In: Cameron N, editor. Human growth and development. San Diego: Academic Press; 2002 pp 165–196.

[108] Blackwell AD, Pryor G, Pozo J, Tiwia W, Sugiyama LS. Growth and Market Integration in Amazonia: A Comparison of Growth Indicators Between Shuar, Shiwiar, and Nonindigenous School Children. Am J Hum Biol. 2009; 21: 161–171. 10.1002/ajhb.20838 18949770 PMC7027596

[109] Smith TM, Reid DJ, Sirianni J E. The accuracy of histological assessments of dental development and age at death. J. Anat. 2006; 208: 125–138. 10.1111/j.1469-7580.2006.00500.x 16420385 PMC2100178

[110] Schillaci MA, Sachdev HPS, Bhargava SK. Technical note: comparison of the Maresh reference data with the WHO International Standard for normal growth in healthy children. Am J Phys Anthropol. 2012;147: 493–498. 10.1002/ajpa.22018 22282150

[111] Orr CM, Dufour DL, Patton JQ. A comparison of anthropometric indices of nutritional status in Tukanoan and Achuar Amerindians. Am J Hum Biol. 2001;13: 301–309. 10.1002/ajhb.1053 11460895

[112] Eerkens JW, Bartelink EJ. Sex-biased weaning and early childhood diet among middle Holocene hunter-gatherers in central California. Am J Phys Anthropol. 2013; 152: 471–483. 10.1002/ajpa.22384 24127159

[113] Sania A, Spiegelman D, Rich-Edwards J, Okuma J, Kisenge R, Msamanga G, et al The contribution of preterm birth and intrauterine growth restriction to infant mortality in Tanzania. Paediatr Perinat Epidemiol. 2014; 8:23–31. 10.1111/ppe.12085 PMC7893612 24117986

[114] Beaumont J, Geber J, Powers N, Wilson AS, Lee-Thorp J, Montgomery J. Victims and survivors: Stable isotopes used to identify migrants from the Great Irish Famine to 19^th^ century London. Am J Phys Anthropol. 2013; 150(1): 87–98. 10.1002/ajpa.22179 23124593

[115] Beaumont J, Atkins EC, Buckberry J, Haydock H, Horne P, Howcroft R, et al Comparing apples and oranges: Why infant bone collagen may not reflect dietary intake in the same way as dentine collagen. Am J Phys Anthropol. 2018; 167(3): 524–540. 10.1002/ajpa.23682 30187451 PMC6221104

[116] Beaumont J, Montgomery J. The Great Irish Famine: Identifying Starvation in the Tissues of Victims Using Stable Isotope Analysis of Bone and Incremental Dentine Collagen. PLoS ONE. 2016; 11(8), e0160065 10.1371/journal.pone.0160065 27508412 PMC4980051

[117] Burt NM. Individual dietary patterns during childhood: an archaeological application of a stable isotope microsampling method for tooth dentin. J Archaeol Sci. 2015; 53: 277–290.

[118] Patrucco R, Tello R, Bonavia D. Parasitological studies of coprolites of prehispanic Peruvian populations. Curr Anthropol. 1983; 24: 393–394.

[119] Huss HH, Reilly A, Ben-Embarek PK. Prevention and control of hazards in seafood. Food Control. 2000; 11:149–156.

[120] Hoover KC, Matsumura H. Temporal variation and interaction between nutritional and developmental instability in prehistoric Japanese populations. Am J Phys Anthropol. 2008; 137: 469–478. 10.1002/ajpa.20892 18711732

[121] Temple DH. What Can Variation in Stature Reveal About Environmental Differences Between Prehistoric Jomon Foragers? Understanding the Impact of Systemic Stress on Developmental Stability. Am J Hum Biol. 2008; 20: 431–439. 10.1002/ajhb.20756 18348169

[122] Piperata BA, Mattern LMG. Longitudinal Study of Breastfeeding Structure and Women’s Work in the Brazilian Amazon. Am J Phys Anthropol. 2011; 144: 226–237. 10.1002/ajpa.21391 20803569

[123] Nanci A. Ten Cate Histologia Oral. São Paulo: Mosby, Elsevier; 2011.

[124] Ghanim A, Manton D, Bailey D, Mariño R, Morgan M. Risk factors in the occurrence of molar-incisor hypomineralization amongst a group of Iraqi children. Int J Paediatr Dent. 2013; 233:197–206 10.1111/j.1365-263X.2012.01244.x 22646757

[125] Ford D, Seow WK, Kazoullis S, Holcombe T, Newman B. A controlled study of risk factors for enamel hypoplasia in the permanent dentition. Pediatr Dent. 2009; 315: 382–388. 19947132

[126] Goodman A, Armelagos GJ. Factors affecting the distribution of enamel hypoplasias man permanent dentition. Am J Phys Anthropol. 1985; 68: 479–493. 10.1002/ajpa.1330680404 3909823

[127] Berbesque JC, Hoover KC. Frequency and developmental timing of linear enamel hypoplasia defects in Early Archaic Texan hunter-gatherers. PeerJ. 2018; 6, e4367 10.7717/peerj.4367 29456891 PMC5815329

[128] Ortner DJ. Theoretical and methodological issues in paleopathology In: Ortner DJ, Aufderheide AC, editors. Human paleopathology: current syntheses and future options. Washington, DC: Smithsonian Institution Press; 1991; pp. 5–11.

[129] Marklein KE, Leahy RE, Crews DE. In sickness and in death: Assessing frailty in human skeletal remains. Am J Phys Anthropol. 2016; 161(2):208–25. 10.1002/ajpa.23019 27312014

