# Supplementary material for: Child development, physiological stress and survival expectancy in prehistoric fisher-hunter-gatherers from the Jabuticabeira II shell mound, South Coast of Brazil
Source: PLoS One. 2020 Mar 11;15(3):e0229684. doi: 10.1371/journal.pone.0229684 (PMC7065757; doi:10.1371/journal.pone.0229684)
Supplement: S1 File — (DOCX) [file pone.0229684.s001.docx]

**Supporting Information S1 Table**

| Table 1: Individuals and long bone measures used for  Delta of Gini calculations | | | | | | |
| --- | --- | --- | --- | --- | --- | --- |
| Individual | Sex | Age | humerus | radio | femur | tibia |
|  |  |  | max. length | max. length | max. length | max. length |
| B. 34 L2.05 | F | YA | 260 | 194 | 361 | 302 |
| B. 11 L1.25 | M | MA | 288 | 231 | 403 | 334 |
| B. 17 A L1.05 | M | MA | 303 | 240 | 430 | 359 |
| B. 118 L6 | M | YA | 298 | 231 | 402 | 341 |

| Table 2: Formulae of Pomeroy and Stock (2012) | | |
| --- | --- | --- |
| Bone | male | female |
| Femur | 44.803 + (LMF*2.738) +/- 2.627 | 48.340 + (LMF*2.593) +/- 2.295 |
|  | 47.207 + (LBF*2.705) +/- 3.644 | 49.147 + (LBF*2.600) +/- 2.231 |
| Tibia | 52.179 + (LMT*2.995) +/- 2.581 | 55.493 + (LMT*2.836) +/- 1.963 |
|  | 53.354 + (LCMT*2.997) +/- 2.524 | 57.748 + (LCMT*2.800) +/- 2.052 |
| F+T | 44.938 +1.488* (LBF+LMT) +/- 2.391 | 45.995 +1.433* (LBF+LMT) +/- 1.927 |
|  | 44.453 + (LBF*1.293) + (LCMT*1.734) +/- 2.290 | 51.947 + (LBF*1.134) + (LCMT*1.637) +/- 1.992 |
|  | 41.814 + (LMF*1.404) + (LMT*1.643) +/- 2.289 | 48.248 + (LMF*0.945) + (LMT*1.943) +/- 1.844 |
| humerus | 53.771 + (LMU*3.483) +/- 3.522 | 61.228 + (LMU*3.155) +/- 2.975 |
| ulna | 51.273 + (LMUl*4.212) +/- 3.615 | 61.525 + (LMUl*3.762) +/- 3.987 |
| radius | 59.305 + (LMR*4.184) +/- 3.467 | 69.331 + (LMR*3.717) +/- 3.012 |
| Measurements: LMF= maximal length of femur; LBF= bicondilar length of femur; LMT= maximal length of tibia; LCMT=Condilo-maleolar length of tibia; LMFi= maximal length of fibula; LMU= maximal length of humerus; LMUl= maximal length of ulna; LMR = maximal length of radius. | | |

| Table 3: Isotope values in adults from Jabuticabeira II ** | | | | | | | | | | | | |
| --- | --- | --- | --- | --- | --- | --- | --- | --- | --- | --- | --- | --- |
| # | Individual  (Burial B#) | Age at death | Sex | Sample* | Section | Age of sample  Ubelaker 1999 | Isotope Values (‰) | | | | | |
|  |  |  |  |  |  |  | δ^13^Cap | δ^13^Ccol | δ^15^N | C% | N% | C:N |
| 1 | B. 34 L2.05 | YA | F | Tooth 1.3 | crown 1 | 5m – 3y | -5.39 | -10.4 | 18.3 | 37.9 | 14.2 | 2.7 |
|  |  |  |  |  | crown 2 | 3-5y |  | -10.4 | 18.1 | 43.6 | 16.3 | 2.7 |
|  |  |  |  |  | root 1 | 6-8y |  | -10.5 | 18.0 | 39.9 | 14.8 | 2.7 |
|  |  |  |  | bone |  | Adult |  | -10.6 | 18.0 | 40.1 | 14.4 | 3.2 |
| 2 | B.36A L2.05 | MA | M | Tooth 2.2 | crown 1 | 1.5 – 2y | -5.72 | -10.9 | 19.3 | 23.6 | 8.1 | 2.9 |
|  |  |  |  |  | crown 2 | 2–4y | -5.05 | -10.4 | 18.3 | 40.1 | 14.3 | 2.8 |
|  |  |  |  |  | root 1 | 4-7y |  | -10.5 | 18.1 | 41.6 | 14.8 | 2.8 |
|  |  |  |  |  | root 2 | 7-10y |  | -10.6 | 18.5 | 41.2 | 14.5 | 2.8 |
|  |  |  |  | bone |  | Adult |  | -10.5 | 17.7 | 40.5 | 14.3 | 3.3 |
| 3 | B.37 L2.05 | OA | M | bone |  | Adult | -7.9 | -10.8 | 17.9 | 41.7 | 14.9 | 3.3 |
| 4 | B.40 L2.05 | MA | F | Tooth 3.4 | crown | 2-6y | -6.19 | -11.0 | 18.7 | 24.4 | 8.7 | 2.8 |
|  |  |  |  |  | root | 6-11y |  | -10.9 | 18.7 | 41.0 | 14.4 | 2.9 |
|  |  |  |  | bone |  | Adult |  | -11.4 | 17.2 | 37.4 | 13.1 | 3.3 |
| 5 | B.41A L2.05 | YA | M | Tooth 4.6 | crown 1 | 6 – 18m | -5.71 | -10.5 | 19.5 | 37.0 | 13.1 | 2.8 |
|  |  |  |  |  | crown 2 | 1.5 – 3y | -5.34 | -10.3 | 18.2 | 40.1 | 14.2 | 2.8 |
|  |  |  |  |  | root 1 | 3-5y |  | -10.3 | 17.7 | 42.3 | 15.0 | 2.8 |
|  |  |  |  |  | root 2 | 5-8y |  | -10.1 | 18.3 | 40.4 | 14.2 | 2.8 |
|  |  |  |  | bone |  | Adult |  | -10.6 | 17.7 | 41.0 | 14.4 | 3.3 |
| 6 | B.108 L2.05 | MA | F | bone |  | Adult |  | -10.9 | 17.2 | 41.8 | 15.1 | 3.2 |
| 7 | B.110 Perf L2 | YA | M | Tooth 2.2 | crown 1 | 1.5 – 2y | -5.95 | -11.2 | 20.0 | 38.5 | 13.4 | 2.9 |
|  |  |  |  |  | crown 2 | 2 – 4y | -5.53 | -11.1 | 19.0 | 38.6 | 13.5 | 2.9 |
|  |  |  |  |  | root 1 | 4-7y |  | -10.9 | 18.3 | 39.4 | 13.8 | 2.8 |
|  |  |  |  |  | root 2 | 7-10y |  | -11.5 | 17.8 | 37.1 | 12.8 | 2.9 |
|  |  |  |  | bone |  | Adult |  | -10.5 | 17.7 | 37.7 | 13.5 | 3.3 |
| 8 | B.11 L1.25 | MA | M | bone |  | Adult |  | -10.3 | 18.0 | 40.3 | 14.2 | 3.3 |
| 9 | B. 12B L1.25 | YA | M | Tooth 2.2 | crown 1 | 1.5 –2y | -6.34 | -10.6 | 21.6 | 34.2 | 12.0 | 2.9 |
|  |  |  |  |  | crown 2 | 2–4y | -5.88 | -10.8 | 20.1 | 37.7 | 13.1 | 2.9 |
|  |  |  |  |  | root 1 | 4-7y |  | -10.6 | 20.1 | 39.9 | 13.9 | 2.9 |
|  |  |  |  |  | root 2 | 7-10y |  | -10.6 | 20.1 | 39.8 | 13.5 | 3.0 |
| 10 | B.24 L1.1.20 | Adult | M | bone |  | Adult |  | -10.5 | 17.8 | 40.8 | 15.0 | 3.2 |
| 11 | B.14 L1.05 | 18y | F | Tooth 4.6 | crown 1 | 6–18 m | -5.82 | -11.0 | 18.2 | 28.8 | 10.0 | 2.9 |
|  |  |  |  |  | crown 2 | 1.5-3y | -6.31 | -10.9 | 17.0 | 29.3 | 10.2 | 2.9 |
|  |  |  |  |  | root 1 | 3-5y |  | -11.2 | 17.0 | 34.7 | 12.1 | 2.9 |
|  |  |  |  |  | root 2 | 5-8y |  | -11.6 | 17.7 | 37.4 | 13.5 | 2.8 |
| 12 | B.15 L1.05 | MA | M | Tooth 2.3 | crown 1 | 6m–3y | -5.10 | -10.2 | 18.6 | 34.3 | 12.1 | 2.8 |
|  |  |  |  |  | crown 2 | 3-5y | -3.94 | -10.0 | 17.8 | 39.9 | 14.2 | 2.8 |
|  |  |  |  |  | root 1 | 5-8y |  | -10.1 | 17.9 | 37.3 | 13.0 | 2.9 |
|  |  |  |  |  | root 2 | 8–11y |  | -10.6 | 17.7 | 35.2 | 12.2 | 2.9 |
|  |  |  |  | bone |  | Adult |  | -11.4 | 16.7 | 30.4 | 10.5 | 3.4 |
| 13 | B.17A L1.05 | MA | M | Tooth 2.2 | crown 1 | 1.5 –2y | -6.00 | -10.4 | 19.1 | 18.7 | 6.4 | 2.9 |
|  |  |  |  |  | crown 2 | 3–4y | -4.89 | -10.3 | 18.2 | 28.6 | 10.1 | 2.8 |
|  |  |  |  |  | root 1 | 4-7y |  | -10.8 | 17.2 | 28.2 | 9.9 | 2.8 |
|  |  |  |  |  | root 2 | 7-10y |  | -10.9 | 16.9 | 32.7 | 11.5 | 2.8 |
|  |  |  |  | bone |  | Adult |  | -11.1 | 17.5 | 37.5 | 13.4 | 3.3 |
| 14 | B.41 L1 | Adult | M | no data |  |  |  |  |  |  |  |  |
| 15 | B.102 L1.75 | MA | F | bone |  | Adult |  | -15.6 | 13.1 | 42.3 | 15.4 | 3.2 |
| 16 | B.123 L1.80 | MA | M | no data |  |  |  |  |  |  |  |  |
| 17 | B.107 L1.85 | OA | M | bone |  | Adult |  | -10.6 | 17.8 | 41.1 | 14.7 | 3.3 |
| 18 | B.2A L6 | Adult | M | bone |  | Adult |  | -11.8 | 16.9 | 35.3 | 12.5 | 3.3 |
| 19 | B.114 L6 | Adult | Ind | bone |  | Adult |  | -11.3 | 18.4 | 41.8 | 15.1 | 3.2 |
| 20 | B.115B L6 | MA | M | Tooth 2.1 | crown 1 | 6 m – 2y | -6.77 | -11.0 | 18.4 | 29.2 | 10.2 | 2.9 |
|  |  |  |  |  | crown 2 | 2-4y | -8.14 | -10.8 | 17.6 | 39.1 | 13.8 | 2.8 |
|  |  |  |  |  | root 1 | 4-6y |  | -10.5 | 17.8 | 40.1 | 14.1 | 2.8 |
|  |  |  |  |  | root 2 | 6-10y |  | -10.9 | 18.3 | 39.9 | 14.1 | 2.8 |
|  |  |  |  | bone |  | Adult | -8.4 | -11.4 | 17.5 | 41.9 | 15.3 | 3.2 |
| 21 | B.118 L6 | YA | M | bone |  | Adult |  | -11.5 | 17.8 | 41.5 | 14.9 | 3.2 |
| 22 | B.121 L6 | MA | M | bone |  | Adult | -8.2 | -10.9 | 17.3 | 40.4 | 14.3 | 3.3 |
| 23 | B.333A L6 | Adult | F | no data |  |  |  |  |  |  |  |  |
| Analytic Precision | | | | | | | ± 0.08 | ± 0.08 | ± 0.10 | ± 0.10 | ± 0.15 |  |
| m: months; y: years. ** Data previously published by Colonese et al. 2014 and Pezo-Lanfranco et al. 2018; * In brackets: tooth number according to FDI notation. (Ad) adolescent (YA) young adult (MA) middle adult (OA) old adult (Adult) adult without estimated age (Buikstra and Ubelaker 1994). | | | | | | | | | | | | |

| Table 4: Isotope values in juveniles from Jabuticabeira II** | | | | | | | | | | | | | |  |  |
| --- | --- | --- | --- | --- | --- | --- | --- | --- | --- | --- | --- | --- | --- | --- | --- |
| Case # | Individual  (Burial B#) | Age at death | Sex | Sample* | Section | Age of sample  Ubelaker 1999 | | Isotope Values (‰) | | | | | | |  |
|  |  |  |  |  |  |  |  | δ^13^Cap | δ^13^Ccol | δ^15^N | C% | N% | C:N |  |  |
| 1 | B.35A L2.05 | 6±3 m | Und | bone |  | 6±3 m | |  | -11.3 | 19.6 | 40.4 | 14.5 | 2.8 |  |  |
| 2 | B. 41B L2.05 | 9±3 m |  |  |  | 9±3 m | |  | -11.1 | 17.8 | 27.4 | 9.5 | 3.4 |  |  |
| 3 | B.120 L2 | 12±4 m | Und | bone |  | 12±4 m | | -7.82 | -10.1 | 20.4 | 40.2 | 14.5 | 2.8 |  |  |
| 4 | B.35B L2.05 | 4y ±12 m | Und | Tooth 7,5 | crown | 6m *IU*- 1y | | -4.64 | -9.4 | 21.0 | 40.5 | 14.8 | 2.7 |  |  |
|  |  |  |  |  | root | 1-3y | |  | -10.8 | 18.8 | 37.3 | 12.8 | 2.9 |  |  |
|  |  |  |  | bone |  | 4y | | -7.33 | -10.2 | 18.3 | 40.3 | 14.7 | 2.7 |  |  |
|  |  |  |  | bone |  | 4y | |  | -10.8 | 18.3 | 40.6 | 14.3 | 3.3 |  |  |
| 5 | B.101 L2.05 | 4y ±12 m | Und | Tooth 8,5 | crown | 6m *IU*- 1y | |  | -15.9 | 14.1 | 41.3 | 15.2 | 3.2 |  |  |
|  |  |  |  |  | root | 1-3y | |  | -14.4 | 14.1 | 41.4 | 15.1 | 3.2 |  |  |
|  |  |  |  | bone |  | 4y | |  | -15.3 | 13.8 | 40.2 | 15.4 | 3.3 |  |  |
| 6 | B.38A L2.05 | 7y ±24 m | Und | Tooth 8,5 | crown | 6m *IU*- 1y | | -5.15 | -10.0 | 21.5 | 37.4 | 13.5 | 2.8 |  | |
|  |  |  |  |  | root | 1-3y | |  | -10.5 | 19.1 | 37.5 | 13.3 | 2.8 |  | |
|  |  |  |  | bone |  | 7y | | -8.47 | -10.7 | 17.7 | 40.0 | 14.5 | 2.8 |  | |
|  |  |  |  | bone |  | 7y | |  | -11.5 | 17.7 | 41.0 | 14.2 | 3.4 |  | |
| 7 | B.10A L1.25 | 7y ±24 m | Und | Tooth 7,5 | crown | 6m *IU* – 1y | | -6.77 | -10.8 | 22.1 | 40.8 | 14.8 | 2.8 |  |  |
|  |  |  |  |  | root | 1-3y | |  | -11.2 | 19.9 | 29.8 | 10.3 | 2.9 |  |  |
|  |  |  |  | bone |  | 7y | | -7.43 | -10.7 | 18.3 | 40.8 | 14.8 | 2.8 |  |  |
|  |  |  |  | bone |  | 7y | |  | -11.4 | 18.5 | 40.4 | 13.8 | 3.4 |  |  |
| 8 | B.17D1 - L1.05 | 6±3 m? | Und | no data |  |  | |  |  |  |  |  |  |  |  |
| 9 | B.17B L1.05 | 6±3 m? | Und | bone |  | 6±3 m? | | -8.87 | -11.8 | 20.1 | 42.1 | 15.0 | 2.8 |  |  |
| 10 | B.3F L6 | 9±3 m | Und | bone |  | 9±3 m | | -10.54 | -11.9 | 20.5 | 40.4 | 14.5 | 2.8 |  |  |
| 11 | B.115A L6 | 9±3 m | Und | bone |  | 9±3 m | | -9.50 | -12.6 | 19.8 | 40.8 | 14.7 | 2.8 |  |  |
| 12 | B.17D2 L1.05 | 9±3 m? | Und | bone |  | 9±3 m? | |  | -11.5 | 20.2 | 43.0 | 15.6 | 3.2 |  |  |
| 13 | B. FS30 - L1.70 | 12±4 m? | Und | no data |  |  | |  |  |  |  |  |  |  |  |
| 14 | B.17D L1.05 | 12±4 m? | Und | bone |  | 12±4 m? | |  | -11.5 | 20.4 | 40.2 | 14.0 | 3.2 |  |  |
| 15 | B. Bebê L1/L2-T | 12±4 m | Und | bone |  | 12±4 m | | -8.73 | -11.7 | 19.6 | 40.8 | 14.6 | 2.8 |  |  |
| 16 | B.119A L6 | 4y ±12 m | Und | Tooth 6,3 | crown | 5m *IU* – 9m | |  | -11.6 | 20.6 | 41.5 | 14.9 | 3.3 |  |  |
|  |  |  |  |  | root | 9 m – 3y | |  | -10.9 | 18.6 | 41.6 | 14.7 | 3.3 |  |  |
|  |  |  |  | bone |  | 4y | | -9.76 | -11.2 | 17.0 | 41.2 | 15.0 | 2.7 |  |  |
| 17 | B.16B L1.05 | 4y ±12 m | Und | Tooth 7,3 | crown 1 | 5m *IU* – 4m | | -5.08 | -10.6 | 21.5 | 14.6 | 5.9 | 2.5 |  |  |
|  |  |  |  |  | crown 2 | 4 – 9m | |  | -10.1 | 19.4 | 37.5 | 14.0 | 2.7 |  |  |
|  |  |  |  |  | root 1 | 9 m – 3y | |  | -11.1 | 18.2 | 9.7 | 4.1 | 2.4 |  |  |
|  |  |  |  | Tooth 7,5 | crown 1 | 6m *IU* – 5 m | | -5.38 | -10.4 | 21.1 | 39.2 | 14.5 | 2.7 |  |  |
|  |  |  |  |  | crown 2 | 5m – 1y | |  | -10.4 | 18.8 | 39.8 | 14.8 | 2.7 |  |  |
|  |  |  |  |  | root 1 | 1-3y | |  | -13.7 | 17.7 | 33.6 | 10.5 | 3.2 |  |  |
|  |  |  |  | bone |  | 4y | | -8.28 | -10.7 | 17.2 | 45.7 | 17.1 | 2.7 |  |  |
|  |  |  |  | bone |  | 4y | |  | -11.2 | 17.4 | 43.5 | 15.4 | 3.3 |  |  |
| 18 | B.104A L1.85 | 15y±36m | Und | Tooth 3,6 | crown 1 | 6–18m | | -5.81 | -10.2 | 19.6 | 35.4 | 12.8 | 2.8 |  |  |
|  |  |  |  |  | crown 2 | 18m-3y | | -5.51 | -10.8 | 17.1 | 34.3 | 12.4 | 2.8 |  |  |
|  |  |  |  |  | root 1 | 3-5y | |  | -11.6 | 16.6 | 35.0 | 11.9 | 2.9 |  |  |
|  |  |  |  |  | root 2 | 5-8y | |  | -12.0 | 17.1 | 34.1 | 11.4 | 3.0 |  |  |
|  |  |  |  | bone |  | 15y | |  | -11.2 | 18.8 | 32.0 | 10.9 | 3.4 |  |  |
| Analytic Precision | | | | | | | ± 0.08 | | ± 0.08 | ± 0.10 | ± 0.10 | ± 0.15 |  |  |  |
| m: months; y: years. ** Data previously published by Colonese et al. 2014 and Pezo-Lanfranco et al. 2018; * tooth number according to FDI notation. (Ad) adolescent (YA) young adult (MA) middle adult (OA) old adult (Adult) adult without estimated age (Buikstra and Ubelaker 1994). | | | | | | | | | | | | | |  |  |

| Table 5: Structure of the sample | | | | | | |
| --- | --- | --- | --- | --- | --- | --- |
| Age | | n | % | Sex | | |
|  |  |  |  | M | F | U |
| Juveniles | Neo | 11 | 26,8 |  |  | 11 |
|  | Inf |  |  |  |  |  |
|  | Ch | 6 | 14,6 |  |  | 6 |
|  | Ad | 1 | 2,4 |  |  | 1 |
| Adults | YA | 7 | 17,1 | 4 | 3 |  |
|  | MA | 8 | 19,5 | 6 | 2 |  |
|  | OA | 3 | 7,3 | 3 |  |  |
|  | Adult | 5 | 12,2 | 3 | 2 |  |
|  | Total | 41 | 100,0 | 16 | 7 | 18 |
| Age categories: Neo: Neonate, 0-1 yr; Inf: Infant, >1-3 yrs; Ch: Child, 4 - 12 yrs; Ad: Adolescent 13-19 yrs; YA: Young Adult, 20 - 35 yrs; MA: Middle Adult, 35 – 50 yrs; OA: old Adult, >50 yrs. Adult: adult without known age. Sex categories: M: males; F: females; U: undetermined sex. | | | | | | |

| Table 6: Teeth with LEH by individual evaluated | | | | | | |
| --- | --- | --- | --- | --- | --- | --- |
|  | N° | Individual | Sex | Age at death | Teeth with hypoplasia | # LEH  (age-range) |
| Adults | 1 | SEP 34 L2.05 | F | YA | UC | 2 (4.0 - 4.8) |
|  | 2 | SEP 36A L2.05 | M | YA | UI1; UC; LC; LI2 | 6 (2.6 - 4.9) |
|  | 3 | SEP 37 L2.05 | M | OA | UI2 UI1; LC | 6 (2.5 - 4.8) |
|  | 4 | SEP 40 L2.05 | F | Adult | UI1; UC; LC | 7 (2.2 - 5.6) |
|  | 5 | SEP 41A L2.05 | M | Adult | UC | 2 (4.0 - 4.8) |
|  | 6 | SEP 108 L2.05 | F | MA | no hypoplasia | no hypoplasia |
|  | 7 | SEP 110 prof L2 | M | YA | UI2; LC; LI2; LI1 | 6 (1.5 - 5.1) |
|  | 8 | SEP 11 L1.25 | M | MA | UI2; UI1; LC | 3 (2.4 - 5.6) |
|  | 9 | SEP 12B L1.25 | M | YA | no hypoplasia | no hypoplasia |
|  | 10 | SEP 24 L1-1.20 | M | Adult |  | nr |
|  | 11 | SEP 14 L1.05 | F | YA | UC | 2 (4.0 - 4.3) |
|  | 12 | SEP 15A L1.05 | M | MA | LC | 2 (2.1 - 4.9) |
|  | 13 | SEP 17 A L1.05 | M | MA | no hypoplasia | no hypoplasia |
|  | 14 | SEP 41 L1 | M | Adult |  | nr |
|  | 15 | SEP 102 L1.75 | F | Adult | no hypoplasia | no hypoplasia |
|  | 16 | SEP 123 L1.80 | M | MA |  | nr |
|  | 17 | SEP 107 L1-T1 | M | OA | no hypoplasia | no hypoplasia |
|  | 18 | SEP 2A L6 | F | MA | no hypoplasia | no hypoplasia |
|  | 19 | SEP 114 L6 | M | OA | UC | 2 (4.4 - 4.8) |
|  | 20 | SEP 115B L6 | M | MA | UC | 2 (4.0 - 4.8) |
|  | 21 | SEP 118 L6 | M | YA | UI1; UI2; UC; LC | 7 (2.9 - 5.6) |
|  | 22 | SEP 121 L6 | M | MA | LC | 2 (3.7 - 4.9) |
|  | 23 | SEP 333A L6 | F | YA |  | nr |
| Juveniles | 1 | SEP 35A - L2.05 | U | 6±3 m |  | nr |
|  | 2 | SEP 17D1 - L1.05 | U | 6±3 m? |  | nr |
|  | 3 | SEP 17B- L1.05 | U | 6±3 m? |  | nr |
|  | 4 | SEP 41B - L2.05 | U | 9±3 m |  | nr |
|  | 5 | SEP 3F - L6 | U | 9±3 m |  | nr |
|  | 6 | SEP 115A-L6 | U | 9±3 m |  | nr |
|  | 7 | SEP 17D2 - L1.05 | U | 9±3 m? |  | nr |
|  | 8 | SEP 120 - L2.05 | U | 12±4 m |  | nr |
|  | 9 | SEP FS30 - L1.70 | U | 12±4 m? |  | nr |
|  | 10 | SEP 17D - L1.05 | U | 12±4 m? |  | nr |
|  | 11 | SEP BEBÊ - L1/L2 | U | 12±4 m |  | nr |
|  | 12 | SEP 35B - L2.05 | U | 4yr±12 m | UI1; LI1 | 2 (2.2 - 3.7) |
|  | 13 | SEP 101- L2.05 | U | 4yr±12 m | UI2; UI1 | 3 (2.8 - 4.0) |
|  | 14 | SEP 119A-L6 | U | 4yr±12 m |  | nr |
|  | 15 | SEP 16B - L1.05 | U | 4yr±12 m | no hypoplasia | no hypoplasia |
|  | 16 | SEP 38 - L2.05 | U | 7yr±24 m | LC (non eruptioned) | 2 (3.7 - 5.6) |
|  | 17 | SEP 10A- L1.25 | U | 7yr±24 m | no hypoplasia | no hypoplasia |
|  | 18 | SEP 104A - L1.85 | U | 15yr±36m | UI2; UC; LC | 6 (2.8 - 5.6) |
|  | | | | | | |
