# Supplementary material for: Child development, physiological stress and survival expectancy in prehistoric fisher-hunter-gatherers from the Jabuticabeira II shell mound, South Coast of Brazil
Source: PLoS One. 2020 Mar 11;15(3):e0229684. doi: 10.1371/journal.pone.0229684 (PMC7065757; doi:10.1371/journal.pone.0229684)
Supplement: S2 File — (DOCX) [file pone.0229684.s002.docx]

| **Supporting Information S2**  Table 1: Timing of LEH in Jabuticabeira II (pooled sample)*, in years. | | | | | |
| --- | --- | --- | --- | --- | --- |
|  | N | Min. | Max. | Mean | SD |
| First LEH | 17 | 1.50 | 4.00 | 3.02 | 0.83 |
| Last LEH | 17 | 3.70 | 5.60 | 4.90 | 0.52 |
| LEH incidence period (time between) | 17 | 0.30 | 3.60 | 1.87 | 1.10 |
| Weaning – 1^st^ LEH period | 16 | -2.00 | 2.00 | 0.57 | 1.05 |
| *only individuals with LEH were considered for calculations. | | | | | |

| Table 2: Timing of LEH in Jabuticabeira II (by sex). | | | | | | |
| --- | --- | --- | --- | --- | --- | --- |
| Sex | | N | Min. | Max. | Mean | SD |
| Male | First LEH | 10 | 1.50 | 4.00 | 2.97 | 0.90 |
|  | Last LEH | 10 | 4.80 | 5.60 | 5.02 | 0.31 |
|  | LEH incidence period (time between) | 10 | 0.80 | 3.60 | 2.05 | 1.06 |
|  | Weaning – 1^st^ LEH period | 10 | -2.00 | 2.00 | 0.47 | 1.17 |
| Female | First LEH | 3 | 2.20 | 4.00 | 3.40 | 1.03 |
|  | Last LEH | 3 | 4.30 | 5.60 | 4.90 | 0.65 |
|  | LEH incidence period (time between) | 3 | 0.30 | 3.40 | 1.50 | 1.66 |
|  | Weaning – 1^st^ LEH period | 3 | 0.20 | 2.00 | 1.23 | 0.92 |

| Table 3: Comparisons of timing of LEH between sexes in Jabuticabeira II | | | | |
| --- | --- | --- | --- | --- |
|  | First  LEH | Last  LEH | LEH incidence period | Weaning – 1^st^ LEH period |
| Mann-Whitney U | 11.000 | 11.000 | 10.500 | 26.000 |
| Wilcoxon W | 66.000 | 17.000 | 16.500 | 54.000 |
| Z | -.696 | -.704 | -.772 | -1.683 |
| Asymp. Sig. (2-tailed) | .487 | .482 | .440 | .101 |

| Table 4: Mean height (in cm) in individuals with and without LEH by sex in Jabuticabeira II | | | | | | |
| --- | --- | --- | --- | --- | --- | --- |
|  | Sex | N | Min. | Max. | Mean | SD |
| Without LEH | M | 3 | 154.30 | 161.17 | 157.56 | 3.44 |
|  | F | 4 | 147.02 | 156.79 | 151.52 | 4.02 |
| With LEH | M | 10 | 150.12 | 166.83 | 156.25 | 5.53 |
|  | F | 3 | 141.08 | 151.35 | 145.12 | 5.47 |
| Males: Mann Whitney U = 12.00 p=0.612; Females: Mann Whitney U = 2.00 p=0.157. | | | | | | |

| Table 5: Comparisons between mean height in individuals with and without LEH from Jabuticabeira II (pooled sample by sex) | | |
| --- | --- | --- |
| Sex | | Height |
| M | Mann-Whitney U | 12.000 |
|  | Wilcoxon W | 67.000 |
|  | Z | -.507 |
|  | Asymp. Sig. (2-tailed) | .612 |
| F | Mann-Whitney U | 2.000 |
|  | Wilcoxon W | 8.000 |
|  | Z | -1.414 |
|  | Asymp. Sig. (2-tailed) | .157 |

**Supporting Information III**

**Logistic Regression Analyses in Adults**

**Model 1: Infant Malnutrition (weaning age < 2 years old) + Infant Morbidity (presence of LEH) as predictors of Adult Stunting (Z-score < 0). Hierarchical model**

| **Case Processing Summary** | | | |
| --- | --- | --- | --- |
| Unweighted Cases^a^ | | N | Percent |
| Selected Cases | Included in Analysis | 20 | 87,0 |
|  | Missing Cases | 3 | 13,0 |
|  | Total | 23 | 100,0 |
| Unselected Cases | | 0 | ,0 |
| Total | | 23 | 100,0 |
| a. If weight is in effect, see classification table for the total number of cases. | | | |

| **Dependent Variable Encoding** | | |
| --- | --- | --- |
| Original Value | | Internal Value |
|  | no stunting | 0 |
|  | stunting | 1 |

**Block 0: Beginning Block**

| **Classification Table^a,b^** | | | | | |
| --- | --- | --- | --- | --- | --- |
|  | Observed | | Predicted | | |
|  |  |  | Adult Stunting (z-score < 0) | | Percentage Correct |
|  |  |  | no stunting | stunting |  |
| Step 0 | Adult Stunting (z-score < 0) | no stunting | 0 | 8 | ,0 |
|  |  | stunting | 0 | 12 | 100,0 |
|  | Overall Percentage | |  |  | 60,0 |
| a. Constant is included in the model.  b. The cut value is ,500 | | | | | |

| **Variables in the Equation** | | | | | | | |
| --- | --- | --- | --- | --- | --- | --- | --- |
|  | | B | S.E. | Wald | df | Sig. | Exp(B) |
| Step 0 | Constant | ,405 | ,456 | ,789 | 1 | ,374 | 1,500 |

| **Variables not in the Equation** | | | | | |
| --- | --- | --- | --- | --- | --- |
|  | | | Score | df | Sig. |
| Step 0 | Variables | Infant malnutrition | 3,333 | 1 | ,068 |
|  | Overall Statistics | | 3,333 | 1 | ,068 |

**Block 2: Method = Enter**

| **Omnibus Tests of Model Coefficients** | | | | |
| --- | --- | --- | --- | --- |
|  | | Chi-square | df | Sig. |
| Step 1 | Step | ,037 | 1 | ,848 |
|  | Block | ,037 | 1 | ,848 |
|  | Model | ,037 | 1 | ,848 |

| **Model Summary** | | | |
| --- | --- | --- | --- |
| Step | -2 Log likelihood | Cox & Snell R Square | Nagelkerke R Square |
| 1 | 26,884^a^ | ,002 | ,002 |
| a. Estimation terminated at iteration number 3 because parameter estimates changed by less than ,001. | | | |

| **Classification Table^a^** | | | | | |
| --- | --- | --- | --- | --- | --- |
|  | Observed | | Predicted | | |
|  |  |  | Adult Stunting (z-score < 0) | | Percentage Correct |
|  |  |  | no stunting | stunting |  |
| Step 1 | Adult Stunting (z-score < 0) | no stunting | 0 | 8 | ,0 |
|  |  | stunting | 0 | 12 | 100,0 |
|  | Overall Percentage | |  |  | 60,0 |
| a. The cut value is ,500 | | | | | |

| **Variables in the Equation** | | | | | | | |
| --- | --- | --- | --- | --- | --- | --- | --- |
|  | | B | S.E. | Wald | df | Sig. | Exp(B) |
| Step 1^a^ | Juvenile morbidity (LEH) | ,182 | ,953 | ,037 | 1 | ,848 | 1,200 |
|  | Constant | ,288 | ,764 | ,142 | 1 | ,706 | 1,333 |
| a. Variable(s) entered on step 1: Juvenile morbidity (LEH). | | | | | | | |

**Model 2: Adult Morbidity (presence of stress markers) as predictor of Early Adult Mortality (< 35 years)**

**Not-hierarchical model. Adults with undetermined age were excluded from analysis.**

| **Case Processing Summary** | | | |
| --- | --- | --- | --- |
| Unweighted Cases^a^ | | N | Percent |
| Selected Cases | Included in Analysis | 18 | 78,3 |
|  | Missing Cases | 5 | 21,7 |
|  | Total | 23 | 100,0 |
| Unselected Cases | | 0 | ,0 |
| Total | | 23 | 100,0 |
| a. If weight is in effect, see classification table for the total number of cases. | | | |

| **Dependent Variable Encoding** | | |
| --- | --- | --- |
| Original Value | | Internal Value |
|  | MA+OA | 0 |
|  | YA | 1 |

**Block 0: Beginning Block**

| **Classification Table^a,b^** | | | | | |
| --- | --- | --- | --- | --- | --- |
|  | Observed | | Predicted | | |
|  |  |  | Age at death < 35 yrs | | Percentage Correct |
|  |  |  | no | yes |  |
| Step 0 | Age at death < 35 yrs | no | 11 | 0 | 100,0 |
|  |  | yes | 7 | 0 | ,0 |
|  | Overall Percentage | |  |  | 61,1 |
| a. Constant is included in the model.  b. The cut value is ,500 | | | | | |

| **Variables in the Equation** | | | | | | | |
| --- | --- | --- | --- | --- | --- | --- | --- |
|  | | B | S.E. | Wald | df | Sig. | Exp(B) |
| Step 0 | Constant | -,452 | ,483 | ,874 | 1 | ,350 | ,636 |

| **Variables not in the Equation** | | | | | |
| --- | --- | --- | --- | --- | --- |
|  | | | Score | df | Sig. |
| Step 0 | Variables | Adult Morbidity | 3,536 | 1 | ,060 |
|  | Overall Statistics | | 3,536 | 1 | ,060 |

**Model 3: Infant Malnutrition (weaning age < 2 years old) + Infant Morbidity (presence of LEH) + Adult Stunting (z-score < 0) as predictors of Early Adult Mortality (< 35 years)**

| **Case Processing Summary** | | | |
| --- | --- | --- | --- |
| Unweighted Cases^a^ | | N | Percent |
| Selected Cases | Included in Analysis | 20 | 87,0 |
|  | Missing Cases | 3 | 13,0 |
|  | Total | 23 | 100,0 |
| Unselected Cases | | 0 | ,0 |
| Total | | 23 | 100,0 |
| a. If weight is in effect, see classification table for the total number of cases. | | | |

| **Dependent Variable Encoding** | | |
| --- | --- | --- |
| Original Value | | Internal Value |
|  | MA+OA | 0 |
|  | YA | 1 |

**Block 0: Beginning Block**

| **Classification Table^a,b^** | | | | | |
| --- | --- | --- | --- | --- | --- |
|  | Observed | | Predicted | | |
|  |  |  | Age at death < 35 yrs | | Percentage Correct |
|  |  |  | no | yes |  |
| Step 0 | Age at death < 35 yrs | no | 12 | 0 | 100,0 |
|  |  | yes | 8 | 0 | ,0 |
|  | Overall Percentage | |  |  | 60,0 |
| a. Constant is included in the model.  b. The cut value is ,500 | | | | | |

| **Variables in the Equation** | | | | | | | |
| --- | --- | --- | --- | --- | --- | --- | --- |
|  | | B | S.E. | Wald | df | Sig. | Exp(B) |
| Step 0 | Constant | -,405 | ,456 | ,789 | 1 | ,374 | ,667 |

| **Variables not in the Equation** | | | | | |
| --- | --- | --- | --- | --- | --- |
|  | | | Score | df | Sig. |
| Step 0 | Variables | Adult Stunting (z-score < 0) | 4,201 | 1 | ,040 |
|  | Overall Statistics | | 4,201 | 1 | ,040 |

**Block 1: Method = Forward Stepwise (Likelihood Ratio)**

| **Omnibus Tests of Model Coefficients** | | | | |
| --- | --- | --- | --- | --- |
|  | | Chi-square | df | Sig. |
| Step 1 | Step | 4,592 | 1 | ,032 |
|  | Block | 4,592 | 1 | ,032 |
|  | Model | 4,592 | 1 | ,032 |

| **Model Summary** | | | |
| --- | --- | --- | --- |
| Step | -2 Log likelihood | Cox & Snell R Square | Nagelkerke R Square |
| 1 | 22,329^a^ | ,205 | ,277 |
| a. Estimation terminated at iteration number 5 because parameter estimates changed by less than ,001. | | | |

| **Hosmer and Lemeshow Test** | | | |
| --- | --- | --- | --- |
| Step | Chi-square | df | Sig. |
| 1 | ,000 | 0 | . |

| **Classification Table^a^** | | | | | |
| --- | --- | --- | --- | --- | --- |
|  | Observed | | Predicted | | |
|  |  |  | Age at death < 35 yrs | | Percentage Correct |
|  |  |  | no | yes |  |
| Step 1 | Age at death < 35 yrs | no | 7 | 5 | 58,3 |
|  |  | yes | 1 | 7 | 87,5 |
|  | Overall Percentage | |  |  | 70,0 |
| a. The cut value is ,500 | | | | | |

| **Variables in the Equation** | | | | | | | | | |
| --- | --- | --- | --- | --- | --- | --- | --- | --- | --- |
|  | | B | S.E. | Wald | df | Sig. | Exp(B) | 95% C.I.for EXP(B) | |
|  |  |  |  |  |  |  |  | Lower | Upper |
| Step 1^a^ | Adult Stunting (z-score < 0) | 2,282 | 1,219 | 3,506 | 1 | ,061 | 9,800 | ,899 | 106,845 |
|  | Constant | -1,946 | 1,069 | 3,313 | 1 | ,069 | ,143 |  |  |
| a. Variable(s) entered on step 1: Adult Stunting (z-score < 0) | | | | | | | | | |

**Block 2: Method = Enter**

| **Omnibus Tests of Model Coefficients** | | | | |
| --- | --- | --- | --- | --- |
|  | | Chi-square | df | Sig. |
| Step 1 | Step | ,170 | 1 | ,680 |
|  | Block | ,170 | 1 | ,680 |
|  | Model | 4,762 | 2 | ,092 |

| **Model Summary** | | | |
| --- | --- | --- | --- |
| Step | -2 Log likelihood | Cox & Snell R Square | Nagelkerke R Square |
| 1 | 22,159^a^ | ,212 | ,286 |
| a. Estimation terminated at iteration number 5 because parameter estimates changed by less than ,001. | | | |

| **Hosmer and Lemeshow Test** | | | |
| --- | --- | --- | --- |
| Step | Chi-square | df | Sig. |
| 1 | ,000 | 1 | 1,000 |

| **Classification Table^a^** | | | | | | | | | | | | |  |  |  |
| --- | --- | --- | --- | --- | --- | --- | --- | --- | --- | --- | --- | --- | --- | --- | --- |
|  | | Observed | | | | Predicted | | | | | | |  |  |  |
|  | |  |  |  |  | Age at death < 35 yrs | | | | Percentage Correct | | |  |  |  |
|  | |  |  |  |  | no | | yes | |  |  |  |  |  |  |
| Step 1 | | Age at death < 35 yrs | no | | | 7 | | 5 | | 58,3 | | |  |  |  |
|  |  |  | yes | | | 1 | | 7 | | 87,5 | | |  |  |  |
|  |  | Overall Percentage | | | |  | |  | | 70,0 | | |  |  |  |
| a. The cut value is ,500 | | | | | | | | | | | | |  |  |  |
| **Variables in the Equation** | | | | | | | | | | | | | | | |
|  | | | | B | S.E. | | Wald | | df | | Sig. | Exp(B) | | 95% C.I.for EXP(B) | |
|  |  |  |  |  |  |  |  |  |  |  |  |  |  | Lower | Upper |
| Step 1^a^ | Adult Stunting (z-score < 0) | | | 2,457 | 1,295 | | 3,601 | | 1 | | ,058 | 11,667 | | ,922 | 147,563 |
|  | Malnutrition | | | -,511 | 1,238 | | ,170 | | 1 | | ,680 | ,600 | | ,053 | 6,795 |
|  | Constant | | | -1,946 | 1,069 | | 3,313 | | 1 | | ,069 | ,143 | |  |  |
| a. Variable(s) entered on step 1: malnutrition. | | | | | | | | | | | | | | | |

**Block 3: Method = Enter**

| **Omnibus Tests of Model Coefficients** | | | | |
| --- | --- | --- | --- | --- |
|  | | Chi-square | df | Sig. |
| Step 1 | Step | 1,202 | 1 | ,273 |
|  | Block | 1,202 | 1 | ,273 |
|  | Model | 5,964 | 3 | ,113 |

| **Model Summary** | | | |
| --- | --- | --- | --- |
| Step | -2 Log likelihood | Cox & Snell R Square | Nagelkerke R Square |
| 1 | 20,957^a^ | ,258 | ,349 |
| a. Estimation terminated at iteration number 5 because parameter estimates changed by less than ,001. | | | |

| **Hosmer and Lemeshow Test** | | | |
| --- | --- | --- | --- |
| Step | Chi-square | df | Sig. |
| 1 | ,225 | 3 | ,973 |

| **Classification Table^a^** | | | | | |
| --- | --- | --- | --- | --- | --- |
|  | Observed | | Predicted | | |
|  |  |  | Age at death < 35 yrs | | Percentage Correct |
|  |  |  | no | yes |  |
| Step 1 | Age at death < 35 yrs | no | 9 | 3 | 75,0 |
|  |  | yes | 3 | 5 | 62,5 |
|  | Overall Percentage | |  |  | 70,0 |
| a. The cut value is ,500 | | | | | |

| **Variables in the Equation** | | | | | | | | | |
| --- | --- | --- | --- | --- | --- | --- | --- | --- | --- |
|  | | B | S.E. | Wald | df | Sig. | Exp(B) | 95% C.I.for EXP(B) | |
|  |  |  |  |  |  |  |  | Lower | Upper |
| Step 1^a^ | Adult Stunting (z-score < 0) | 2,869 | 1,467 | 3,824 | 1 | ,051 | 17,622 | ,994 | 312,515 |
|  | Malnutrition | -1,296 | 1,513 | ,734 | 1 | ,391 | ,274 | ,014 | 5,305 |
|  | Juvenile morbidity (LEH) | 1,437 | 1,393 | 1,064 | 1 | ,302 | 4,210 | ,274 | 64,629 |
|  | Constant | -3,010 | 1,599 | 3,545 | 1 | ,060 | ,049 |  |  |
| a. Variable(s) entered on step 1: Juvenile morbidity (LEH). | | | | | | | | | |

**Model 4: Adult Stunting (z-score < 0) as predictor of Early Adult Mortality (< 35 years).**

| **Case Processing Summary** | | | |
| --- | --- | --- | --- |
| Unweighted Cases^a^ | | N | Percent |
| Selected Cases | Included in Analysis | 23 | 100,0 |
|  | Missing Cases | 0 | ,0 |
|  | Total | 23 | 100,0 |
| Unselected Cases | | 0 | ,0 |
| Total | | 23 | 100,0 |
| a. If weight is in effect, see classification table for the total number of cases. | | | |

| **Dependent Variable Encoding** | | |
| --- | --- | --- |
| Original Value | | Internal Value |
|  | MA+OA | 0 |
|  | YA | 1 |

**Block 0: Beginning Block**

| **Classification Table^a,b^** | | | | | |
| --- | --- | --- | --- | --- | --- |
|  | Observed | | Predicted | | |
|  |  |  | Age at death < 35 yrs | | Percentage Correct |
|  |  |  | no | yes |  |
| Step 0 | Age at death < 35 yrs | no | 13 | 0 | 100,0 |
|  |  | yes | 10 | 0 | ,0 |
|  | Overall Percentage | |  |  | 56,5 |
| a. Constant is included in the model.  b. The cut value is ,500 | | | | | |

| **Variables in the Equation** | | | | | | | |
| --- | --- | --- | --- | --- | --- | --- | --- |
|  | | B | S.E. | Wald | df | Sig. | Exp(B) |
| Step 0 | Constant | -,262 | ,421 | ,389 | 1 | ,533 | ,769 |

| **Variables not in the Equation** | | | | | |
| --- | --- | --- | --- | --- | --- |
|  | | | Score | df | Sig. |
| Step 0 | Variables | Adult Stunting (z-score < 0) | 6,303 | 1 | ,012 |
|  | Overall Statistics | | 6,303 | 1 | ,012 |

**Block 1: Method = Forward Stepwise (Likelihood Ratio)**

| **Omnibus Tests of Model Coefficients** | | | | |
| --- | --- | --- | --- | --- |
|  | | Chi-square | df | Sig. |
| Step 1 | Step | 6,964 | 1 | ,008 |
|  | Block | 6,964 | 1 | ,008 |
|  | Model | 6,964 | 1 | ,008 |

| **Model Summary** | | | |
| --- | --- | --- | --- |
| Step | -2 Log likelihood | Cox & Snell R Square | Nagelkerke R Square |
| 1 | 24,528^a^ | ,261 | ,350 |
| a. Estimation terminated at iteration number 5 because parameter estimates changed by less than ,001. | | | |

| **Hosmer and Lemeshow Test** | | | |
| --- | --- | --- | --- |
| Step | Chi-square | df | Sig. |
| 1 | ,000 | 0 | . |

| **Classification Table^a^** | | | | | |
| --- | --- | --- | --- | --- | --- |
|  | Observed | | Predicted | | |
|  |  |  | Age at death < 35 yrs | | Percentage Correct |
|  |  |  | no | yes |  |
| Step 1 | Age at death < 35 yrs | no | 8 | 5 | 61,5 |
|  |  | yes | 1 | 9 | 90,0 |
|  | Overall Percentage | |  |  | 73,9 |
| a. The cut value is ,500 | | | | | |

| **Variables in the Equation** | | | | | | | | | |
| --- | --- | --- | --- | --- | --- | --- | --- | --- | --- |
|  | | B | S.E. | Wald | df | Sig. | Exp(B) | 95% C.I.for EXP(B) | |
|  |  |  |  |  |  |  |  | Lower | Upper |
| Step 1^a^ | Adult Stunting (z-score < 0) | 2,667 | 1,198 | 4,954 | 1 | ,026 | 14,400 | 1,375 | 150,808 |
|  | Constant | -2,079 | 1,061 | 3,844 | 1 | ,050 | ,125 |  |  |
| a. Variable(s) entered on step 1: Stunting (Zscore < 0) | | | | | | | | | |

**Logistic Regression Analyses in Juveniles**

**Model 1: Infant Morbidity + Stunting as predictors of Early Juvenile Mortality <1 year (hierarchical model)**

| **Case Processing Summary** | | | | | | |
| --- | --- | --- | --- | --- | --- | --- |
| Unweighted Cases^a^ | | | | | N | Percent |
| Selected Cases | | | Included in Analysis | | 18 | 100,0 |
|  |  |  | Missing Cases | | 0 | ,0 |
|  |  |  | Total | | 18 | 100,0 |
| Unselected Cases | | | | | 0 | ,0 |
| Total | | | | | 18 | 100,0 |
| a. If weight is in effect, see classification table for the total number of cases. | | | | | | |
| **Dependent Variable Encoding** | | | |  |  |  |
| Original Value | | Internal Value | |  |  |  |
|  | >1 yr | 0 | |  |  |  |
|  | <1 yr | 1 | |  |  |  |

| **Categorical Variables Codings** | | | |
| --- | --- | --- | --- |
|  | | Frequency | Parameter coding |
|  |  |  | (1) |
| Stunting | no | 7 | ,000 |
|  | yes | 11 | 1,000 |
| Infant Morbidity | no | 8 | ,000 |
|  | yes | 10 | 1,000 |

**Block 0: Beginning Block**

| **Classification Table^a,b^** | | | | | |
| --- | --- | --- | --- | --- | --- |
|  | Observed | | Predicted | | |
|  |  |  | Juvenile Mortality <1yr | | Percentage Correct |
|  |  |  | yes | no |  |
| Step 0 | Juvenile Mortality <1yr | yes | 11 | 0 | 100,0 |
|  |  | no | 7 | 0 | ,0 |
|  | Overall Percentage | |  |  | 61,1 |
| a. Constant is included in the model.  b. The cut value is ,500 | | | | | |

| **Variables in the Equation** | | | | | | | |
| --- | --- | --- | --- | --- | --- | --- | --- |
|  | | B | S.E. | Wald | df | Sig. | Exp(B) |
| Step 0 | Constant | ,452 | ,483 | ,874 | 1 | ,350 | 1,571 |

| **Variables not in the Equation** | | | | | |
| --- | --- | --- | --- | --- | --- |
|  | | | Score | df | Sig. |
| Step 0 | Variables | Infant Morbidity | 1,169 | 1 | ,280 |
|  | Overall Statistics | | 1,169 | 1 | ,280 |

**Model 2: Infant Morbidity + Stunting as predictors of Early Juvenile Mortality <1 year (not-hierarchical model).**

| **Case Processing Summary** | | | |
| --- | --- | --- | --- |
| Unweighted Cases^a^ | | N | Percent |
| Selected Cases | Included in Analysis | 18 | 100,0 |
|  | Missing Cases | 0 | ,0 |
|  | Total | 18 | 100,0 |
| Unselected Cases | | 0 | ,0 |
| Total | | 18 | 100,0 |
| a. If weight is in effect, see classification table for the total number of cases. | | | |

| **Dependent Variable Encoding** | | |
| --- | --- | --- |
| Original Value | | Internal Value |
|  | >1 yr | 0 |
|  | <1 yr | 1 |

| **Categorical Variables Codings** | | | |
| --- | --- | --- | --- |
|  | | Frequency | Parameter coding |
|  |  |  | (1) |
| Stunting | no | 7 | ,000 |
|  | yes | 11 | 1,000 |
| Infant Morbidity | no | 8 | ,000 |
|  | yes | 10 | 1,000 |

**Block 0: Beginning Block**

| **Classification Table^a,b^** | | | | | |
| --- | --- | --- | --- | --- | --- |
|  | Observed | | Predicted | | |
|  |  |  | Juvenile Mortality <1yr | | Percentage Correct |
|  |  |  | yes | no |  |
| Step 0 | Juvenile Mortality <1yr | yes | 11 | 0 | 100,0 |
|  |  | no | 7 | 0 | ,0 |
|  | Overall Percentage | |  |  | 61,1 |
| a. Constant is included in the model.  b. The cut value is ,500 | | | | | |

| **Variables in the Equation** | | | | | | | |
| --- | --- | --- | --- | --- | --- | --- | --- |
|  | | B | S.E. | Wald | df | Sig. | Exp(B) |
| Step 0 | Constant | ,452 | ,483 | ,874 | 1 | ,350 | 1,571 |

| **Variables not in the Equation** | | | | | |
| --- | --- | --- | --- | --- | --- |
|  | | | Score | df | Sig. |
| Step 0 | Variables | Juvenile morbidity (1) | 1,169 | 1 | ,280 |
|  |  | Stunting (1) | ,513 | 1 | ,474 |
|  | Overall Statistics | | 1,425 | 2 | ,490 |
